# A zoological catalogue of hunted reptiles in the semiarid region of Brazil

**DOI:** 10.1186/1746-4269-8-27

**Published:** 2012-07-30

**Authors:** Rômulo Romeu Nóbrega Alves, Gentil Alves Pereira Filho, Kleber Silva Vieira, Wedson Medeiros Silva Souto, Lívia Emanuelle Tavares Mendonça, PauloFernandoGuedesPereira Montenegro, Waltécio de Oliveira Almeida, Washington Luiz Silva Vieira

**Affiliations:** 1 Departamento de Biologia, Universidade Estadual da Paraíba, Av. das Baraúnas, 351/Campus Universitário, Bodocongó, 58109-753, Campina Grande-PB, Brazil; 2 Laboratório de Ecofisiologia Animal, Departamento de Sistemática e Ecologia da Universidade Federal da Paraíba, 58051-900, João Pessoa, PB, Brazil; 3 Programa de Pós-Graduação em Ciências Biológicas (Zoologia), Laboratório de Ecofisiologia Animal, Departamento de Sistemática e Ecologia da Universidade Federal da Paraíba, 58051-900, João Pessoa, PB, Brazil; 4 Departamento de Química Biológica, Bolsista Produtividade da Fundação Cearense de Apoio ao Desenvolvimento Científico e Tecnológico — FUNCAP, Universidade Regional do Cariri, Rua Cel. Antônio Luiz 1161, CEP, 63100-000, Crato, CE, Brazil

## Abstract

The variety of interactions between human cultures and herpetofauna is the subject matter of Ethnoherpetology, a subdivision of Ethnozoology. In the semi-arid region of Brazil, many reptiles interact with human communities because of their utility or because of the risks they represent. These interactions have obvious implications for the conservation of reptiles from this region.In this context, ethnoherpetology studies are crucial because they serve as subsidies for guiding strategies for the handling and conservation of reptiles. This paper presents ethnozoological and taxonomic informations of hunted reptiles in the semiarid region of Brazil and analyse the implications on conservation that are related to the interactions between people and reptiles in this region. Taxonomic keys to identifying recorded reptiles are provided. Records of humans interacting with 38 reptile species that belong to 31 genuses and 16 families have been found. The groups with the largest numbers of recorded species were snakes (18 species), and this group was followed in number by lizards (13), chelonians (4), and crocodilians (3). The reptiles that were recorded may be used for the following purposes: medicinal purposes (24 species), food (13 species), ornamental or decorative purposes (11 species), in magical/religious practices (10 species), and as pets (10 species). Some species (n = 16) may have multiple uses. Furthermore, more than half of the species (n = 19) are commonly killed because they are considered potentially dangerous. Strategies for conserving the reptiles of the Brazilian semi-arid region must reconcile and integrate human and conservation needs.

## Introduction

Brazil has occupied the second position in the list of countries with greater richness of reptile species, only behind Australia (with 864 recorded species, according to Wilson and Swan [1], but surpassing Mexico, India, Indonesia, Colombia, China, and Peru [2]. There are currently 732 species of reptiles known in Brazil, of which 690 belong to the Squamata (375 serpents, 248 lizards and 67 amphisbaenids); there are also six species of caimans and 36 species of turtles [2]. A significant part of Brazilian herpetofauna has been used by traditional human populations, and some are still used by modern societies [3]. Many stories, myths and proverbs have been generated from these relationships and also have been passed from generation to generation through oral traditions, influencing how local people relate to these animals [4-7].

Products derived from reptiles (including leather, teeth, fat, meat and bones) have nutritional, ornamental and medicinal values in many rural and urban areas in Brazil and these animals are often sought after as pets and zoological attractions [3].The Caatinga represents one of the major examples of a semi-arid environment in the Neotropical region, where it is a biome that is extremely threatened due to the unsustainable use of natural resources. In this biome, 117 species of reptiles are recorded (7 Testudines, 47 lizards, 10 Amphisbaenia, 52 serpents, and 3 alligators) [8-10]. Local human populations have interacted with many of these species by attributing some utility value to them. Additionally, some species are hunted and killed due to conflicting relations with people [3,11-13]. The main reasons for the conflicts, which lead to the killing of reptiles, include attacks on livestock and risk to human lives.

The cultural richness of the local population and its diverse interactions with the local fauna make the Caatinga an advantageous area for ethnozoological studies. These factors are fundamentally important within a socio-environmental perspective because excessive exploitation, hunting and illegal trades of wild animals are threats to some species of vertebrates of this biome [11]. Nonetheless, over the past few decades, researchers have begun to systematically investigate the relationship between local inhabitants and the wild fauna of this region. Ethnoherpetology is a subdivision of Ethnozoology that examines the relationships between human cultures and herpetofauna [3,14-16]. Very few ethnoherpetological studies have been undertaken in Brazil [4,13,17-22], which restricts our ability to elaborate adequate conservation strategies for many species [3,23].

In the last few years, the importance of the ethnobiological studies for the biodiversity conservation has been increasingly recognized [11,24-27], which is not surprising due the strong human influence on the biodiversity. Native or local people retain a wide range of biological information than can complement traditional academic knowledge in zoology, ecology, and biological conservation studies [28-31]. In the case of animal conservation, it is evidenced that the perception and use ways of animals by humans are extremely relevant to the definition of possible conservation strategies. Hence, an understanding of the cultural, social, and traditional roles of the fauna is essential for establishing management plans directed towards sustainable use [3].

In the specific case of reptiles, a recent review revealed that only five studies on ethnoherpetology were performed in the Brazilian semiarid region [32], although some ethnozoological studies have certified the use of reptiles in the region for mainly medicinal purposes [33-41]. When analysing the current panorama, it is evident that new ethnoherpetological studies are required in Caatinga, especially due to their importance in supporting management plans for the local herpetofauna. A recent ethnozoological review by Alves and Souto [32] noted that ethnozoological research in Brazil has grown quantitatively, but these authors also highlighted that there is a clear need for qualitative improvements in the generated publications. Among the aspects that must be improved, authors have found that those studies should have a greater taxonomic accuracy, which is an aspect that is missed in most of the ethnozoological studies that have been performed. Many of the articles are based only on lists of species, which are often taxonomically incorrect or are restricted to only the popular names of the animals. In this context, the present study aims to accomplish the following: i) to elaborate a catalogue of the reptiles of ethnozoological importance from the Brazilian semi-arid region; ii) to present a brief characterisation of each species, while aiming to support new studies of ethnoherpetology in this region; iii) to present an analysis of the relationship between the local people and some native reptiles, while focusing on the utilitarian value that these human groups assign to these animals and on the conflicting relationships that are associated with this zoological group; and iv) to analyse the implications on conservation that are related to the interactions between people and reptiles in this semi-arid region of Brazil.

## Methods

### Study area

**Figure 1:**
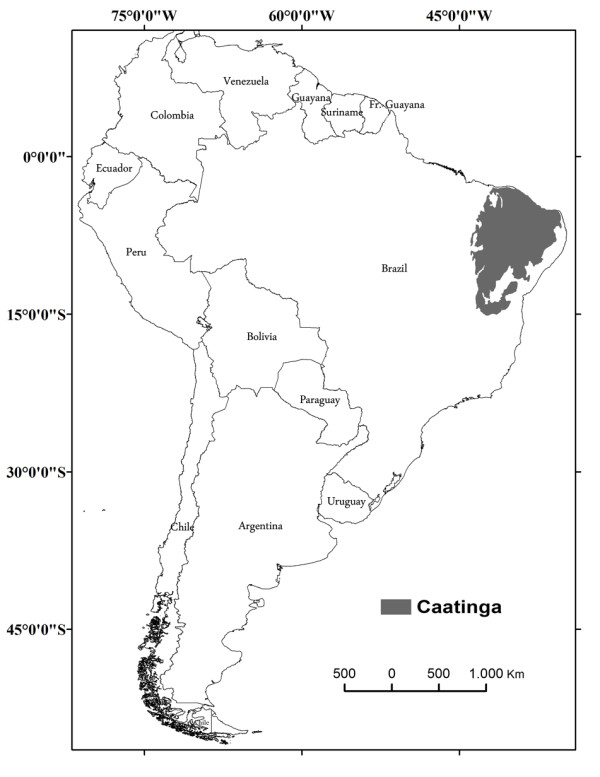
Map showing the area of Caatingain semiarid region of Brazi.

The Brazilian semi-arid region occupies an area of approximately 1 million km² (Figure 1) that is mainly characterized by an average annual rainfall of less than 800 mm, high potential evapotranspiration and an aridity index of 0.5 or a drought risk of more than 60% [42,43].The predominant vegetation type of semiarid region of Brazil is composed of several forms of caatinga biome [44]. The structure of these forests can vary considerably from forests composed of mostly spiny trees, 6 to 10 m tall, often with a ground-layer of small deciduous shrubs and annual herbs, predominantly Leguminosae, to deciduous woodlands of lower stature, with a high proportion of shrubs and subshrubs and the presence of many cacti, bromeliads and Euphorbiaceae [45].

The Caatinga has been described as harboring relatively few species and having low numbers of endemic species [46-48]. Some recent studies, however, have challenged this and demonstrated the importance of the region for the conservation of a significant component of Brazilian biodiversity [49]. Inventories and assessments have, to date, recorded 932 vascular plant species [50], 187 bees [51], 244 fishes [52,53], 175 reptiles and amphibians [8,53], 62 families and 511 species of birds [53,54], and 156 mammal species [53,55]. Levels of endemism vary from about 7% for mammals [55] to 3% in birds [54] and 57% in fishes [52].

### Data collection

To examine reptilian species that are involved in relationships with local people within the semiarid region of Brazil, we reviewed references and reports. Only taxa that could be identified to the species level were included in the database, and the scientific nomenclature of the species that are cited in this study follows the guidelines of the *Brazilian Society of Herpetology* (http://www.sbherpetologia.org.br/). The conservation status of the reptilian species follows IUCN [56] and the Brazilian Red List [57], and a database was created that contains information on the species of the reptiles, their uses and their ways of interacting with people.

In addition, information have been gotten through visiting several rural and urban areas in the semi-arid region in the states of Paraíba and Pernambuco, where the authors have performed several studies of zoological and ethnozoological purposes. In these areas have been carried out interviews with hunters and traders of products from wild animals. It has been interviewed 436 hunters in different cities from the semi-arid region and 49 traders in public market places distributed in Campina Grande (state of Paraíba), Caruaru and Santa Cruz do Capibaribe (state of Pernambuco). From these interviews, it has been recorded the potential species of reptiles of utility value and their respective uses, or whether there was any conflict relationship with human populations. The ethical approval for the study was obtained from the Ethics committee of Paraiba University State (N° of protocol: 0026.0.133.000-10).

The species identification was made by individuals collected by researchers over the field work and by specimens donated spontaneously by local residents. Animals were collected with the permission of the Instituto Chico Mendes de Conservação da Biodiversidade (ICMBio) and the Sistema de Autorização e Informação em Biodiversidade (SISBIO), license numbers 25926-2and 14105–2. Specimens collected were housed at the herpetological collection of the Universidade Federal da Paraíba, Brazil.

## Results

Our review revealed that various methods of interactions between people and reptiles in the semi-arid region of Brazil have developed and that such interactions are associated with the utility value of the species or are a result of the conflicting relationships that are associated with this zoological group. Additionally, many species have become a source of tales, myths, beliefs and fables in this region. Records of humans interacting with at least 38 reptile species that belong to 31 genuses and 16 families have been found. The groups with the largest numbers of recorded species were snakes (18 species), and this group was followed in number by lizards (13), chelonians (4), and crocodilians (3) (Table 1).

**Table 1:** Reptile species, their respective uses, conflicting characteristics in the semiarid region of Brazil, and status of conservation

**Family/ Species**	**Uses andConflicting relationships**						**IUCN RedList**
	**F**	**M**	**MR**	**P**	**O**	**CR**	
**CAYMANS**							
**Alligatoridae**							
*Caiman crocodilus* (Linnaeus, 1758)	▴	▴	▴	▴	▴	▴	LC
*Caiman latirostris* (Daudin, 1802)	▴	▴	▴	▴	▴	▴	LC
*Paleosuchus palpebrosus* (Cuvier, 1807)	▴	▴	▴	▴	▴	▴	LC
**SNAKES**							
**Boidae**							
*Boa constrictor* Linnaeus, 1758	▴	▴	▴	▴	▴	▴	
*Corallus hortulanus* (Linnaeus, 1758)		▴				▴	
*Epicrates assisi* Machado, 1945		▴		▴		▴	
**Colubridae**							
*Drymarchon corais* (Boie, 1827)							
*Leptophis ahaetulla* (Linnaeus, 1758)		▴					
*Oxybelis aeneus* (Wagler, 1824)						▴	
*Spilotes pullatus* (Linnaeus, 1758)		▴	▴			▴	
*Tantilla melanocephala* (Linnaeus, 1758)						▴	
**Dipsadidae**							
*Boiruna sertaneja* Zaher, 1996						▴	
*Liophis viridis* Günther, 1862						▴	LC
*Oxyrhopus trigeminus* Duméril, Bibron & Duméril, 1854		▴	▴			▴	
*Philodryas nattereri* Steindachner, 1870			▴				
*Philodryas olfersii* (Lichtenstein, 1823)						▴	
*Pseudoboa nigra* (Duméril, Bibron & Duméril, 1854)						▴	
*Xenodon merremii* (Wagler, 1824)			▴				
**Elapidae**							
*Micrurus ibiboboca* (Merrem, 1820)		▴				▴	
**Viperidae**							
*Bothropoides erythromelas* (Amaral, 1923)						▴	
*Crotalus durissus* (Linnaeus, 1758)	▴	▴	▴		▴	▴	LC
**CHELONIAN**							
**Chelidae**							
*Mesoclemmys tuberculata* (Lüderwaldt, 1926)	▴	▴		▴			
*Phrynops tuberosus* (Peters, 1870)	▴	▴		▴	▴		
**Kinosternidae**							
*Kinosternon scorpioides* (Linnaeus, 1766)	▴	▴					NT
**Testudinidae**							
*Chelonoidis carbonaria* (Spix, 1824)	▴	▴	▴	▴	▴		
**LIZARDS**							
**Amphisbaenidae**							
*Amphisbaena alba* Linnaeus, 1758					▴		LC
*Amphisbaena polystega* (Duméril, 1851)						▴	LC
*Amphisbaena vermicularis* Wagler, 1824					▴		
**Gekkonidae**							
*Hemidactylus mabouia* (Moreau de Jonnès, 1818)		▴					
**Iguanidae**							
*Iguana iguana* (Linnaeus, 1758)	▴	▴		▴			
**Phyllodactylidae**							
*Phyllopezus periosus* Rodrigues, 1986					▴		
*Phyllopezus pollicaris* (Spix, 1825)		▴					
**Polychrotidae**							
*Polychrus acutirostris* (Spix, 1825)		▴					
**Teiidae**							
*Ameiva ameiva* (Linnaeus, 1758)	▴	▴					
*Cnemidophorus ocellifer* (Spix, 1825)	▴	▴					
*Tupinambis merianae* (Duméril & Bibron, 1839)	▴	▴		▴	▴	▴	LC
**Tropiduridae**							
*Tropidurus hispidus* (Spix, 1825)		▴					LC
*Tropidurus semitaeniatus* (Spix, 1825)		▴					
**TOTAL: 38species**							

**Legend:** F- Food resource, M- Medicinal, MR- Magic/religious, P- Pets, O- Ornamentation and decoration, CR- Conflicting relationships. IUCN Red List categories (World Conservation Union; *http://www.iucnredlist.org/*): LC - Least Concern, CD - Conservation dependent, CR - Critically Endangered, EN - Endangered, NT - Near threatened, VU – Vulnerable.

**Figure 2:**
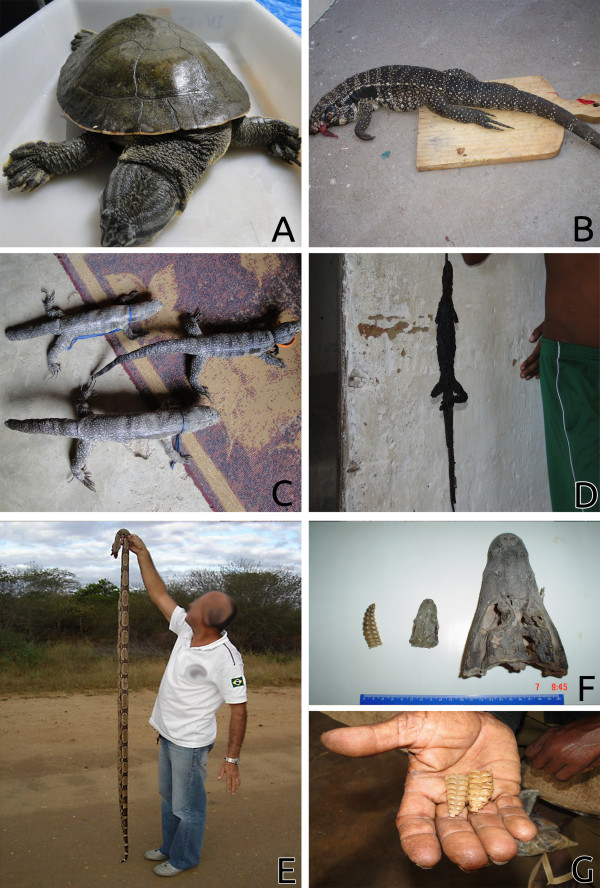
**Examples of interactions between people and reptiles in the semi-arid region of Brazil.A**- *Phrynops tuberosus* to be used as food or formedicinal purposes, *Tupinambis merianae* killed for food (**B**), and subsequently stuffed to be used as an ornament (**C**), *Iguana iguana* being roasted for subsequent medicinal use (**D**), *Boa constrictor* killed by local people (**E**), **F**- rattlesnake´s rattle (*C. durissus*); head of jibóia (*Boa constrictor*) and of caiman (*C. crocodilus*) used for medicinal and magic religious purposes and a rattlesnake rattle used for similar purposes (**G**).

The reptiles that were recorded may be used for the following purposes: medicinal purposes (23 species), food (13 species), ornamental or decorative purposes (11 species), in magical/religious practices (10 species), and as pets (10 species) (Figure 2). Some species (n = 16) may have multiple uses. Furthermore, more than half of the species (n = 19) are commonly killed when they come into contact with humans.

General features of the recorded reptiles and their respective ethnozoological notes are described in the following sections.

### Lizards

#### Teiidae family

**Figure 3:**
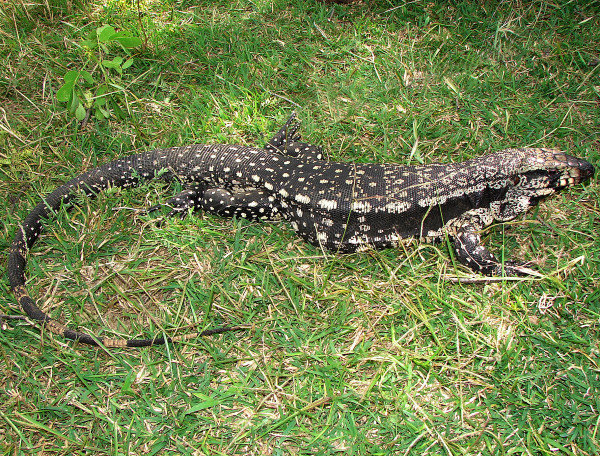
***Tupinambis merianae*(Duméril & Bibron, 1839) - (Photo - Washington Vieira).**

*Tupinambis merianae* (Duméril & Bibron, 1839) (Figure 3) - Common names: Argentine black and white tegu, “téjo”, “tejuaçú”, “teiú”.

Comments: *Tupinambis merianae *is a large-sized lizard that may reach 30 to 50 cm in cloacal rostrum length. The dorsum of the adult is generally black and grey or black and dark brown, marbled colour and can contain transverse stripes that have distinct, randomly spread, white or yellowish spots [46,58] The dorsal region of the head is sooty brown, and in some individuals, the flanks can be a lighter colour, have spots that can be black, and present transverse stripes and a randomly spread or wide, black strip that begins immediately behind the opening of the drum, follows along the body, and continues in a series of bright spots that can vary in their levels of tonality and are margined above and below black [46]. This lizard has a white or cream belly with black spots that vary in size and are randomly distributed.

The frontal portion of the bodies of young specimens is greenish and followed by a series of black, transverse stripes that are interspersed by light-grey or dark-grey stripes [46]. The bodies of these organisms are cylindrical, have a granular dorsal region and large ventral and rectangular scales that are arranged into 28 to 34 transverse rows [46,58]. This species is found in a widely spread region and in a large variety of habitats from the southern Amazon to northern Argentina [46,58].

Ethnozoological notes: Species from the genus *Tupinambis *are most likely the most hunted lizards in South America, especially for meat and leather products, which had great commercial value until the 1980s [59-62]. In the semi-arid region of Brazil, *T. merianeae* represents the main cinegetic reptile. Members of this species are used for their skin, tongue, fat, meat and, sometimes, as pets [33,63,64]. The meat of these animals is used as a source of protein for the local population and may still be used commercially on a small scale in urban areas [11,65]. Marques and Guerreiro [65] have recorded *Tupinambis* spp. being sold in public marketplaces in the city of Feira de Santana, which is located in the state of Bahia. In traditional folk medicine, the fat of *T. merianae* is one of the most widely used zootherapeutic products in Caatinga [33,34,38,66], and this material is used in the treatments of the following ailments: earaches, deafness, rheumatism, erysipelas, skin problems, respiratory illnesses, sore throat, snake bites, asthma, tumours, swelling, infections and bronchitis. Additionally, other medicinal products, such as tongue and skin, are derived from this species. Additionally, medicinal products derived from this species can be used ethnoveterinary medicine [36,39,40,67]. Furthermore, because they are potential predators of poultry and their eggs, members of this species have been hunted due to it causing losses to human populations [11]. *T. merianeae *may be hunted occasionally or in directed hunting expeditions, and it can be killed using firearms and hounds, with the aid of stones and sticks, or using traps [11,12,68]. The cinegetic importance of *T. merianae *and other species of this genus has been recorded in various locations in Brazil [3] and in other countries [59-61]. In some cases, the species is considered as food taboo and it is not consumed, such as in some localities the Brazilian Atlantic Forest [69,70].

**Figure 4:**
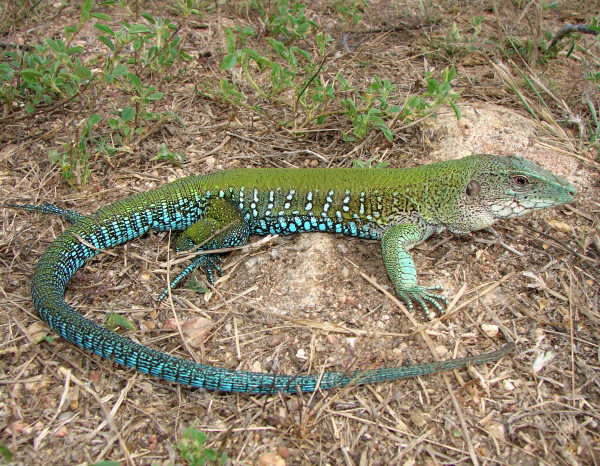
***Ameiva ameiva*(Linnaeus, 1758) (Photo - Washington Vieira).**

*Ameiva ameiva* (Linnaeus, 1758) (Figure 4) - Common names: South American Ground Lizard, “bico doce”, “sardão grande”.

Comments: *Ameiva ameiva* is a medium-sized lizard that can reach 16 to 20 cm in cloacal rostrum length, and the colour pattern of this organism varies with age. Adults may have a completely green back or the anterior region of the body can be light or dark brown with randomly spread cross-links and black spots [46,58,71,72]. The flanks show a similar pattern to the back but have white spots that are surrounded by black and are sometimes distributed into vertical rows. The womb and lower surface of the posterior limbs are an intense blue colour in males [46,58]. The bodies of the young may be colourful or completely brown, or the front part of the body could be green and the flanks could have a black strip that extends from the back of the head to the base of the tail [46,58]. These lizards have a cylindrical body, granular dorsal region, and large ventral, rectangular scales that are arranged into 10 transverse rows. This species is widely spread and is found in the eastern Andes, Panama, and to northern Argentina [46].

Ethnozoological notes: The consumption of this species as food has occurred, but this consumption occurs less extensively than that of *T. merianae*. This species is used in popular medicine to treat infections, dermatitis, venereal diseases, and snake bites [33,64].

**Figure 5:**
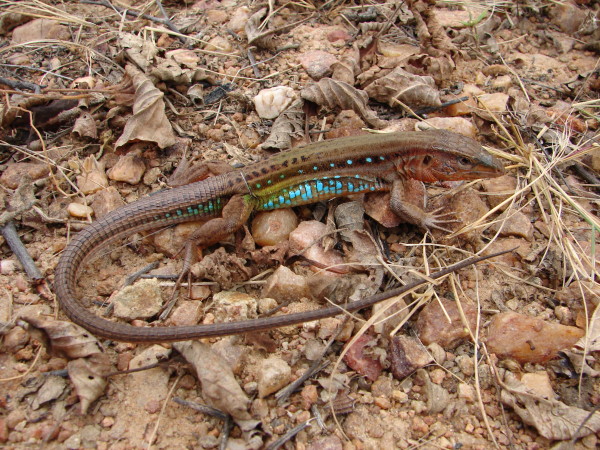
***Cnemidophorus ocellifer*(Spix, 1825) (Photo - Washington Vieira).**

*Cnemidophorus ocellifer* (Spix, 1825) (Figure 5) - Common names:Spix's Whiptail, “calanguinho”, “calango”, “sardão pequeno”.

Comments: *Cnemidophorus ocellifer* is a small-sized lizard that can reach 9 to 12 cm in cloacal rostrum length, and the colour pattern varies with age. Adult males may have a back that varies from greenish to light brown or reddish brown, flanks with dark-brown, irregular, longitudinal lines, and one of the lines could be dotted by round, whitish or bluish spots. The belly can be reddish, bluish or whitish [46]. Subadult males and adult females may present a back with bluish tonality and less clear, light lines that have dark, interrupted stripes, flanks with the same dorsal pattern and a white belly. Young individuals have longitudinal, whitish lines that are very visible and interspersed with dark-brown or light-brown stripes, flanks with the same pattern of the back and a white belly [46]. These lizards have a cylindrical body, a granular dorsal region, and large ventral, rectangular scales that are arranged into 8 transverse rows. *Cnemidophorus* ocellifer is widely spread across South America, and it has been recorded in a variety of habitats [46,73-78]. According to Arias et al. [9,10], the species that is commonly identified as *C. ocellifer * forms a complex, and the taxonomy of the group will be subject to significant changes once new species are described over the next years.

Ethnozoological notes: This small lizard is usually hunted by children who slaughter them for entertainment using "baladeiras" (sling-shots). Sometimes, the captured animals are used as food, and products from this species are used in popular medicine to treat the following diseases: infections, dermatitis, venereal diseases and snakebites [33,64].

#### Iguanidae family

**Figure 6:**
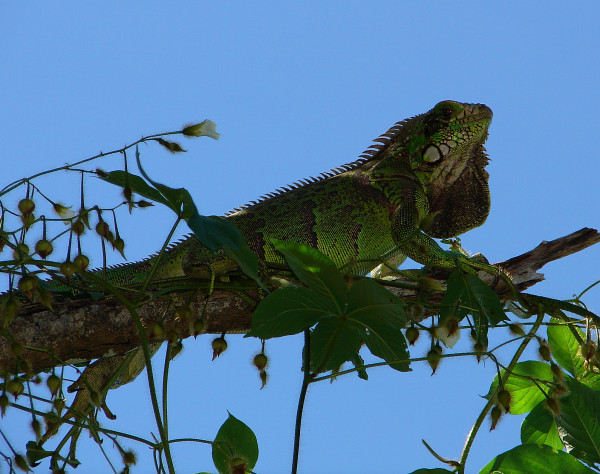
***Iguana iguana*(Linnaeus, 1758) (Photo - Washington Vieira).**

*Iguana iguana* (Linnaeus, 1758) (Figure 6) - Common names: Common Green Iguana, “camaleão”.

Comments: *Iguana iguana* is a large-sized animal that can reach 32 to 40 cm in cloacal rostrum length, and when including its tail, it may reach 1.5 m. The young, immature individuals are green with dark, transverse stripes on their backs, flanks, and tail and have a white belly. The adults are darker and may be completely grey [46,58,71,72]. These animals have a robust body and limbs and have a prominent, continuous vertebral crest that extends from their tail end to nape. They have a short head, round and short muzzle, large and oval tympanum, a large and round scale that is located on the tympanum, a large and appendix or gular crest, and a medial row of triangular scales that are laterally compressed [46]. This species is found in a widely spread area that includes México, Central America, areas of the Antilles, Brazil and Paraguay [46,58,71,72].

Ethnozoological notes: This species represents the second most important species of lizards from the Caatinga; however, unlike *T.merianeae,* it is not hunted much. The meat of this organism has been used as a source of protein, and other products, such as the fat and bones, have been used in local medicine [11]. *I. Iguana *is also ordinarily used as a pet throughout Brazil [3]. The consumption and commercialisation of *I. iguana* meat is common in the tropical Americas [79,80], and its meat and eggs are the principal protein sources of many diets. In Brazil, the consumption of chameleon “camaleões” has been reported in rural areas and in large urban centres [3,65]. Alves et al. [3] have discovered that the meat and eggs of chameleon “camaleões” are widely consumed by members of traditional fishing communities who live on Ilha do Marajó in the Amazon River, which is in the state of Pará in the northern area of Brazil.

#### Polychrotidae family

**Figure 7:**
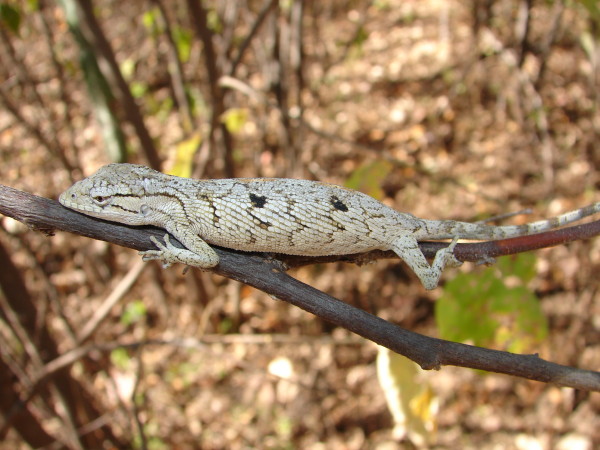
***Polychrus acutirostris*(Spix, 1825) (Photo - Washington Vieira).**

*Polychrus acutirostris* (Spix, 1825) (Figure 7) - Common names: Sharp-nosed monkey lizard, “papa vento”, “calango cego”.

Comments: *Polychrus acutirostris* is a small-sized animal that can reach 14 to 25 cm in cloacal rostrum length. Its back and flanks vary in colour from light to dark grey, light to dark brown, or olive-grey to grey or grey-brown. However, some specimens may be greyish-white with darker spots [46,58,71]. Black lines begin at the eyes, two distinct lines extend to the shoulders, and the lower line passes through the tympanum [46]. The body of this animal is relatively slim and laterally compressed, and it has slim and long limbs. The tail is long and semi-prehensile, it has conical eyes, and its eyelids are partially fused [46,58,71]. This species is found in open-space environments in cis-Andean South America, the southern area of the Pará state in northern Brazil, and to northern Argentina [46,58].

Ethnozoological notes: The species is used for medicinal purposes, and it is locally believed that it may improve sexual strength.

#### Gekkonidae family

**Figure 8:**
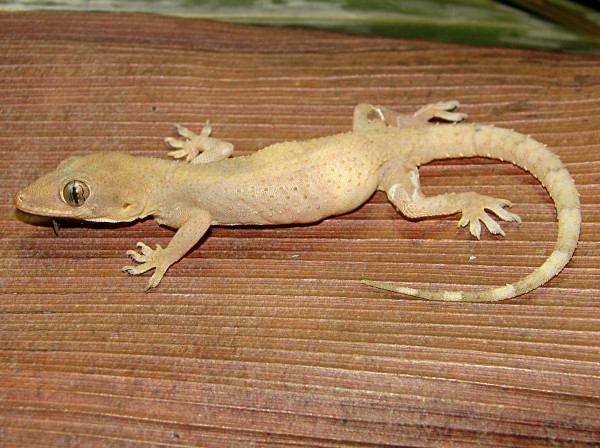
***Hemidactylus mabouia*(Moreau de Jonnès, 1818) (Photo - Washington Vieira).**

*Hemidactylus mabouia* (Moreau de Jonnès, 1818) (Figure 8)- Common names: Afro-american house Gecko, tropical house gecko, “lagartixa”, “lagartixa de parede”, “briba”.

Comments: *Hemidactylus mabouia* is a small-sized animal that may reach 6 to 10 cm in cloacal rostrum length. The colour of its back is variable and ranges from light grey to dark grey or from light brown to dark brown and can sometimes be nearly white. Additionally, it has many dark spots, which can be irregular-shaped or round, and four to six transverse stripes spread along its back [46,58,71,72]. The womb is whitish and may have small dots. The head is flat and large and in the dorsal-ventral position; the eyes are large and have elliptical pupils; and its back has small granules that are interspersed with tubercles that are conical and fairing. The hands and feet of this animal have a double row of lamellae, which are transversally extended. The species is found in Africa and was introduced in America. It has now spread to Central America, the Caribbean, South America and Florida, USA [46,58,71,72]. In Brazil, the first records of *H. mabouia* in natural habitats occurred in 1945 [81].

Ethnozoological notes: *H. mabouia*has been used in popular medicine to treat sore throat.

#### Phyllodactylidae family

**Figure 9:**
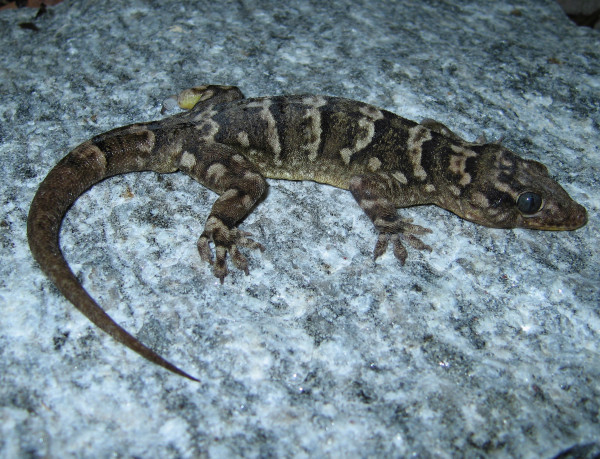
***Phyllopezus periosus*(Rodrigues, 1986) (Photo – Cláudio Sampaio).**

*Phyllopezus periosus* (Rodrigues, 1986) (Figure 9) - Common names: Paraiba Gecko, “briba”.

Comments: *Phyllopezus periosus*is a small-sized animal that may reach 9 to 12 cm of cloacal rostrum length. Its back is greyish and sometimes brown. Usually, 6 to 7 irregular, transverse, reddish-brown or dark-brown spots are found that are separated by light-grey coloured halos, and the lateral and dorsal surfaces of the tail have the same coloured pattern. The bellies of adult specimens are yellowish, but the bellies of young specimens are whitish [82]. The head is large and flat and in the dorsal-ventral position; its eyes are large, without eyelids and with elliptical pupils. Small, smooth and juxtaposed granules that are interspersed by conical tubercles, which are sparse and distinctly taller, are found on the backs of this organism. The digits of the hands and feet have, in their ventral areas, a row of whole and smooth lamellae and some distal and imbricate lamellae [82]. This species is found exclusively in the Caatinga area in northeastern Brazil [82,83].

Ethnozoological notes: This lizard is used in popular medicine.

**Figure 10:**
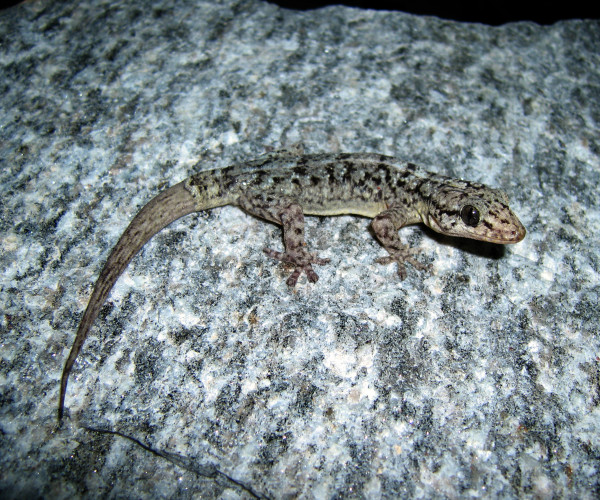
***Phyllopezus pollicaris*(Spix, 1825) (Photo – Cláudio Sampaio).**

*Phyllopezus pollicaris* (Spix, 1825) (Figure 10)- Common names: Brazilian Gecko, and “briba”.

Comments: *Phyllopezus pollicaris *is a small-sized animal that may reach 6 to 9 cm in cloacal rostrum length. Its back colour varies from light to dark grey and has randomly spread black dots. Additionally, transverse dark stripes are occasionally interrupted by a light, medium-dorsal line. Certain adult specimens may be a darker colour, and in these cases, the transverse stripes and the medium-dorsal line are less pronounced. On the side of the organism, there is a dark stripe that spans from the orbit, passes through the tympanum and slightly beyond the shoulder, and reaches the lateral edge of the first three transverse stripes of the back [46,84]. The bellies of adult and young specimens are white, and the head is flat and large and in the dorsal-ventral position. The eyes are large, without eyelids and with elliptical pupils. The back has small granules that are smooth and juxtaposed and are interspersed with larger distal tubercles, which are conical and sparse. The digits of the hands and feet possess a row of whole and smooth lamellae, and in the hands and feet, the distal lamellae are generally imbricate [46,82,84]. The species is found to be widely spread in open spaces of South America, especially in Caatinga, Cerrado and in other Brazilian seasonal dry tropical forests, the Paraguayan chaco, southern Bolivia and northern Argentina [83].

Ethnozoological notes: This lizard is used in popular medicine.

#### Tropiduridae family

**Figure 11:**
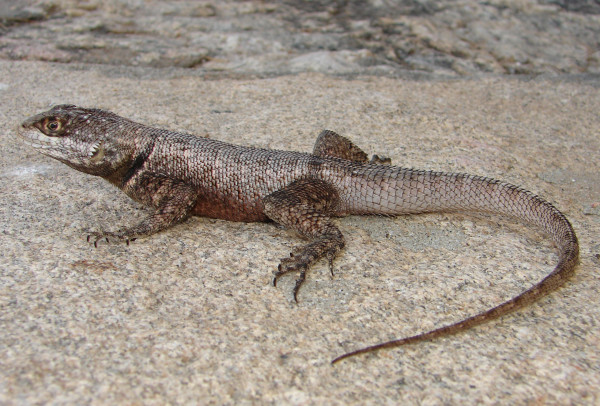
***Tropidurus hispidus*(Spix, 1825)(Photo - Washington Vieira).**

*Tropidurus hispidus* (Spix, 1825) (Figure 11)- Common names: Peters' Lava Lizard, “lagartixa”, “lagartixa-preta”, “catenga”, “catexa”.

Comments: *Tropidurus hispidus* is a small-sized animal that may reach 6 to14 cm in cloacal rostrum length. The colour of its back varies from dark to lighter grey or dark to lighter brown with irregularly shaped, dark-brown, transverse stripes that may be pale or absent in some specimens [85]. A dark-brown, anti-humeral stripe in a semi-collar shape and margined by a light line that may or may not be dorsally united can be present [46,58,85]. The colouring of the belly varies from light to dark grey, and the lower surface of the posterior limbs and the anal tab of adult males is black. The dorsal scales are strongly keeled, bristling, and arranged in oblique rows that converge toward the sacral region. The ventral scales are smaller than those of the dorsal region and are smooth, imbricate, round or rhomboid [46,58,85]. This species is widely spread and can be found in northeastern South America, predominantly in the northeasternCaatinga, in open areas in the northern portion of the Amazon River, and has been introduced around Manaus city in the Central Amazon [72,85].

Ethnozoological notes: This animal has been used in popular medicine to treat alcoholism, dermatomycosis, warts, abscesses, boils, sore throat, erysipelas and to heal the umbilical cord of newborn babies.

**Figure 12:**
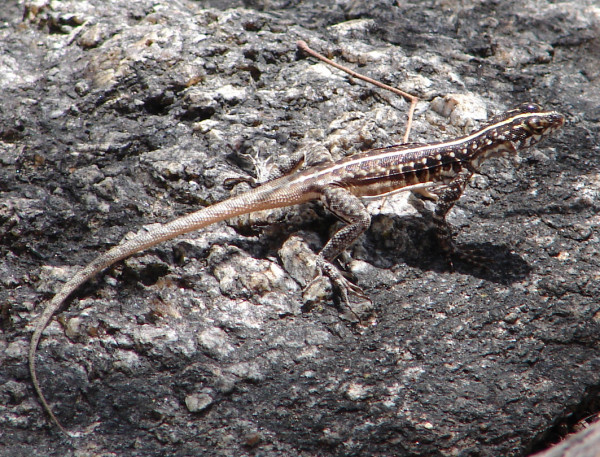
***Tropidurus semitaeniatus*(Spix, 1825) (Photo - Washington Vieira).**

*Tropidurus semitaeniatus* (Spix, 1825) (Figure 12) - Common names: Striped Lava Lizard, “lagartixa-de-lajedo”, “lagartixa”.

Comments: *Tropidurus semitaeniatus* is a small-sized lizard that may reach 5 to 10 cm in cloacal rostrum length. A medial, whitish stripe that spans from the muzzle to the base of the tail on a greyish or brownish background is presented on the backs of adults. In this region, some irregularly arranged dark spots and whitish dots are also observed [46]. Young animals have a whitish, medium-dorsal stripe, which is similar to adults, and white spots, which form transverse lines, on a nearly black background [46]. The ventral area varies from light to dark grey, and the internal surface of the leg and anal tab of adult males is blackened. This organism has a flat body in the dorsoventral position, has small dorsal scales, lacks fins, and has sleek, irregularly shaped and sized ventral scales that are larger than those of the dorsal region [46]. This species is typically found in northeastern Caatinga and is quite common on rocky outcrops; it is also found from Piauí to the northern part of Bahia state, near Salvador city [8,46].

Ethnozoological notes: This animal is used in popular medicine in the treatment of measles, asthma, alcoholism, dermatomycosis and warts [86].

#### Amphisbaenidae family

**Figure 13:**
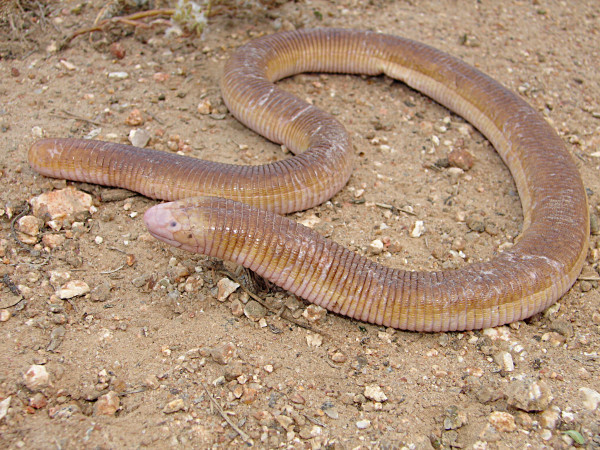
***Amphisbaena alba*(Linnaeus, 1758) (Photo - Washington Vieira).**

*Amphisbaena alba* (Linnaeus, 1758) (Figure 13) - Common names: Redworm Lizard, “cobra-de-duas-cabeças”.

Comments: *Amphisbaena alba* is a medium-sized animal that may reach 70 to 75 cm in cloacal rostrum length. The adults’ colour varies from yellowish to light brown, and it has a whitish belly [87]. This organism has an elongated and cylindrical body, ring-shaped scales, and a short and round tail, which resembles a head. The body has 4 to 10 pre-cloacal pores, and paws are absent. The muzzle is rounded, and the eyes are rudimentary and covered by a scale [71,72,87,88]. This species is widely spread throughout the central eastern area of South America, which contains a variety of phytogeographic features [71,72,87,88].

Ethnozoological notes: This species is usually confused with poisonous snakes, and for this reason, people from local communities commonly kill it.

**Figure 14:**
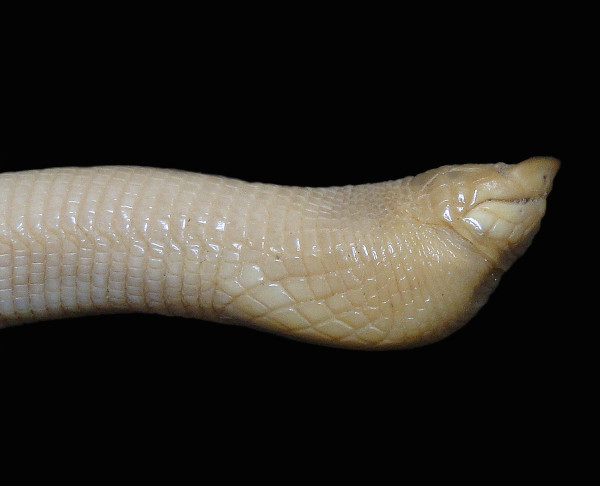
***Amphisbaenapolystega*(Duméril, 1851) (Photo - Washington Vieira)**

*Amphisbaena polystega* (Duméril, 1851) (Figure 14)- Common name: Bahia Worm Lizard, “cobra-de-duas-cabeças”.

Comments**: ***Amphisbaena polystega* is a medium-sized animal that may reach 30 to 35 cm in cloacal rostrum length. The adults’ colour varies from yellowish to light brown and can sometimes be nearly greyish, and the belly is whitish [89]. This animal has an elongated and cylindrical body, ring-shaped scales, a short tail that is rounded on the end, no paws, and two pre-cloacal pores. The head is always dorsoventrally compressed; the snout is broad, slightly upturned and in a lateral profile; the openings of the nostrils are on the ventral surface of the snout; and the scale rostral and nasals are fused into a single rostronasal shield that is followed by a sequence of one to five enlarged shields along the dorsal surface of the head. The eyes are primitive and are covered by a scale, and the pectoral shields are mostly diamond-shaped [89-91]. *A. polystega* is found in central, northern and northeastern Brazil (i.e., in the phytogeographic regions of the Amazon, Atlantic Forest, Caatinga, and Cerrado) [89,91].

Ethnozoological notes: This organism is usually confused with poisonous snakes, and for this reason, people from local communities commonly kill it.

**Figure 15:**
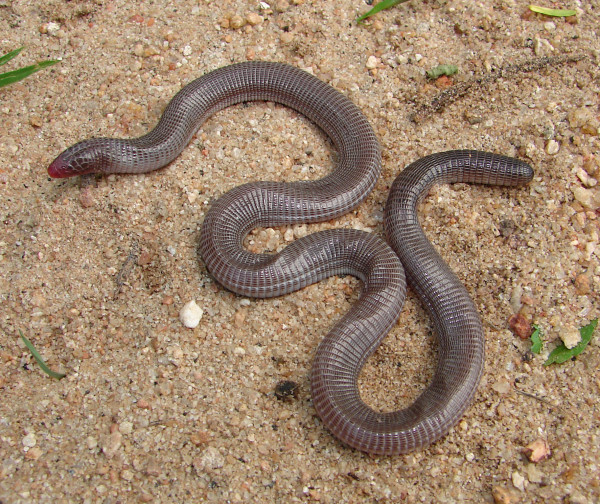
***Amphisbaena vermicularis*(Wagler, 1824) (Photo - Washington Vieira).**

*Amphisbaena vermicularis* (Wagler, 1824) (Figure 15) –Common names: Wagler's Worm Lizard, “cobra-de-duas-cabeças”.

Comments: *Amphisbaena vermicularis* is a small-sized animal that may reach 32 to 35 cm in cloacal rostrum length. The adults’ colour varies from greyish to light brown, and it has a whitish belly [46]. This organism has an elongated and cylindrical body, ring-shaped scales, a short tail that is rounded on the end, and a level of autotomy in the fifth and seventh segment. *A. vermicularis *has four pre-cloacal pores, no paws, a rounded muzzle, and primitive eyes that are covered by a scale [46,88]. This species is widely spread in the northeastern region of Brazil and has been recorded in the Pará, Minas Gerais and Mato Grosso states [46,88].

Ethnozoological notes: This species is usually confused with poisonous snakes, and for this reason, people from local communities commonly kill it.

### Chelonians

#### Chelidae family

**Figure 16:**
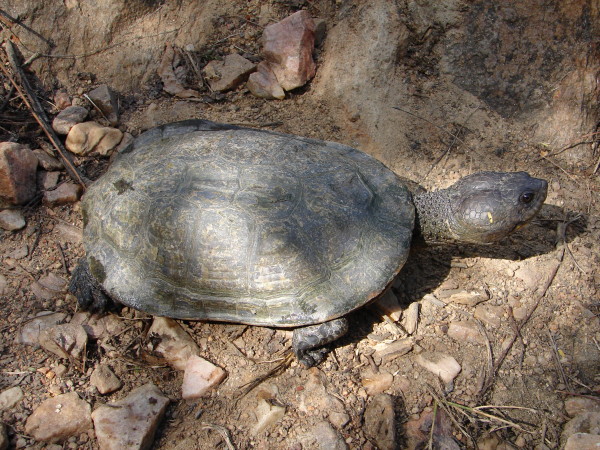
***Mesoclemmys tuberculata*(Lüderwaldt, 1926) (Photo - Washington Vieira).**

*Mesoclemmys tuberculata* (Lüderwaldt, 1926) (Figure 16) - Common names: Tuberculate toad-headed turtle, “cágado”, “cágado-d’água”.

Comments: *Mesoclemmys tuberculata* is a medium-sized species that may reach 25 to 30 cm in rectilinear length of the carapace [46,92,93]. The colouring of the carapace may vary from light to dark brown or can be entirely black. The surface of each scute may be roughened with raised striations [92,94], and the plastron is yellowish and can present blackish spots, which darken completely as the animals age [94]. The head and neck have a completely dark grey back, and the ventral region is yellowish or greyish. The back of the head can be sprinkled with light dots, and the jaws are light yellow with a lighter stripe on the upper region [46,92,94]. The neck is covered with conical tubercles, which gives the species its name [92,94]. This species is widely spread in northeastern Brazil and has been recorded in areas of Caatinga, the Atlantic Forest and Cerrado. It has been found in nearly all northeastern states and in the northern area of the Minas Gerais state [95,96].

Ethnozoological notes: This species is used as food by human populations from the semi-arid region and is used in popular medicine to treat the following illnesses: rheumatism, thrombosis, bronchitis, diarrhoea, bleeding, asthma, sore throat and hoarseness [33,64,97]. Occasionally, this organism may be incidentally captured in fishnets. In this situation, animal may be used or discarded by local people.

**Figure 17:**
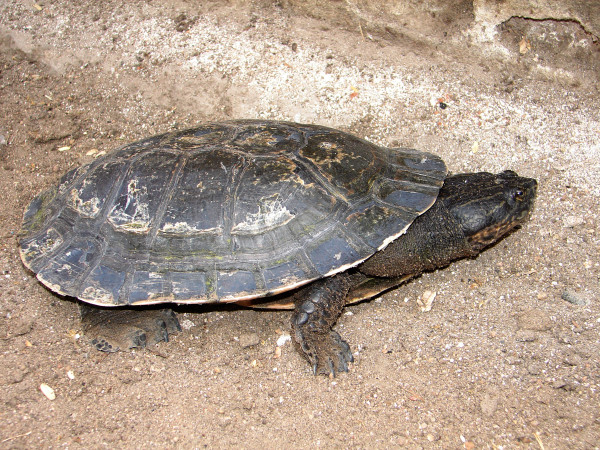
***Phrynops tuberosus*(Peters, 1870) (Photo - Washington Vieira).**

*Phrynops tuberosus* (Peters, 1870) (Figure 17) - Common name: Peters’s side-necked turtle, “cágado”, “cágado-d’água”.

Comments: *Phrynops tuberosus* is a medium-sized species that may reach 27 to 30 cm of rectilinear length of the carapace [46,71,92-94]. Its carapace is dark brown, black with grey mottling, striped by a reticulum of mahogany-coloured streaks and dots, and has a yellow border [71,92]. The plastron, in older specimens, can be uniformly yellow, light brown or yellowish with small, clear, dark spots. Juveniles and young adults have an extensive red and black pattern [46,92]. The head is grey or olive in the dorsal region with a thick, longitudinal, cream stripe that begins at the nostril and ends at the shoulders. This light line is bordered on one side by a black line that begins at the orbit and by another that begins along the jaws [71]. The throat and the ventral portion of the neck are whitish or yellowish with thick, black cross-links that are distributed irregularly [46]. Some specimens may present a clear, median keel that is marked on each side by a series of outgrowths on the costal scutes, which form a discontinuous line. The dorsal surface of the neck can contain small tubercles or fine, long, needle-shaped tubercles [46,92]. The forefront and posterior areas of the plastron are immovable [46,92]. This species is found in Guyana, the southeastern portion of Venezuela, Suriname, French Guyana, the eastern Amazon basin, and in areas of northeastern Brazil [71,92,94].

Ethnozoological notes: This species is used as food and in popular medicine to treat asthma, sore throat, swelling, earache, rheumatism, arthritis, and mumps and to heal the umbilical cords of newborn babies [33,66,98-100]. It may also be incidentally captured in fishnets, when animal may be used or discarded by local people.

#### Kinosternidae family

**Figure 18:**
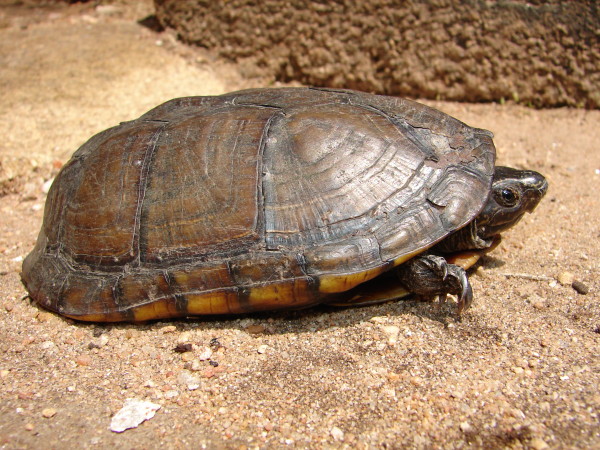
***Kinosternon scorpioides *(Linnaeus, 1766) (Photo - Washington Vieira).**

*Kinosternon scorpioides* (Linnaeus, 1766) (Figure 18) - Common names: Scorpion Mud Turtle, “muçuã”,“cágado”.

Comments: *Kinosternon scorpioides *is a small-sized chelonian that may reach 18 to 27 cm of rectilinear length of the carapace [46,93]. The colour of the carapace can vary from light to dark brown, and the plastron and the lower marginal plaques are yellowish, although they may also have dark brown spots. Its head and neck and the dorsal portions of the back and womb are dark, the head can be sprinkled with light dots, and the jaws are light yellow. The elongated, oval carapace is high-domed; it has three well-developed, longitudinal keels that are developed at several levels and disappears in older animals [92,94]; and the forefront and posterior portions of the plastron are mobile [46,92]. This species is found in southern Tamaulipas, Mexico; Central America; Guyana; Bolivia; Ecuador; Peru; the northern, northeastern and midwestern portions of Brazil; and northern Argentina [46,71,92,93].

Ethnozoological notes: *K. scorpioides *has been used for human consumption and medicinal purposes. These organisms can be incidentally captured in fishnets. In this situation, animal may be used or discarded by local people.

#### Testudinidae family

**Figure 19:**
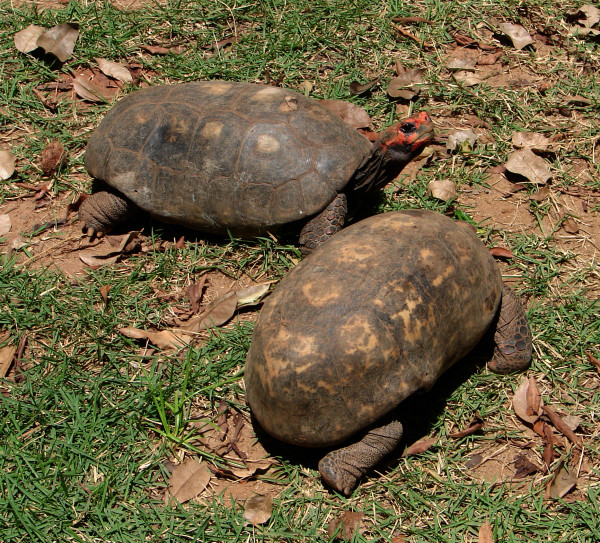
***Chelonoidis carbonaria*(Spix, 1824) (Photo - Washington Vieira).**

*Chelonoidis carbonaria* (Spix, 1824) (Figure 19) - Common names: Red-footed tortoise, “jabuti”.

Comments: *Chelonoidis carbonaria* is a medium-sized species that may reach 30 to 70 cm of rectilinear length of the carapace. Its colour may vary from very dark brown to black, the centre-most area of the scute (areola) is yellow or orange, the plastron varies from yellow to orange, and each scute is outlined along the growth lines by dark pigments. The head of this organism is dark, and it has scales that vary from red to yellow. The back and ventral portions of the neck are completely dark grey. The legs are columnar-shaped, and the anterior portions of the limbs present red or orange scales [92,94]. This chelonian is found in southern Panama, in the western Andes in Colombia Chaco, Venezuela, from Guyana to eastern Brazil, in southern Rio de Janeiro, from western to eastern Bolivia, in Paraguay, and in northern Argentina. It seems to be absent from nearly all eastern areas of the Amazon basin [92-94].

Ethnozoological notes: The meat of the species can be used as food, and its fat is used in popular medicine to treat the following diseases: erysipelas, bronchitis, asthma and to stop the sensation of thirst [33,98,100,101]. This species includes some of the most popular wild animals used as pets in the region because these animals are relatively docile and easy to capture and care for. A popular belief is that their presence helps to prevent illnesses such as bronchitis and asthma [3,4]. According to Fitzgerald [102] and Lopes [103], *Chelonoidis* sp. is the most frequently commercialised reptile in Brazil (and in the world), and it is widely sold to pet shops, private collectors and zoos.

### Snakes

#### Boidae family

**Figure 20:**
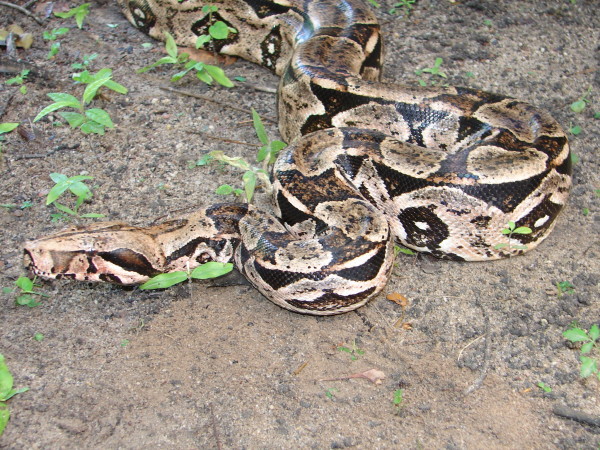
***Boa constrictor*(Linnaeus, 1758) (Photo - Washington Vieira).**

*Boa constrictor* (Linnaeus, 1758) (Figure 20) - Common names: Boa, “jibóia”, “cobra de veado”.

Comments: A medium to large sized snake with a wide distribution in Central and South America [104]. In Brazil, this species can be found in forest and open vegetal formations such as Atlantic Forest and Caatinga [46,76]. The diet is based primarily on mammals like small rodents but sometimes birds and lizards are also consumed. *Boa constrictor* is terrestrial and arboreal and presents nocturnal activity but it is not uncommon find this species active during the day.

Ethnozoological notes: Records of the use of this species as food exist in some locations in the northeastern semi-arid region of Brazil; through interviews with hunters, the consumption of the meat of this species was recorded in Pocinhos city (Paraíba State). The consumption of *B. constrictor* in the city of Pedra Branca, which is located in the state of Bahia, was also reported by Santos-Fita et al. [13]. In addition, *B. constrictor* is widely used in traditional communities for many different types of popular medicine [17,34,37,64,66,105], and products derived from this species are sold in public marketplaces throughout northeastern Brazil [41,86,98,106]. The most widely used zootherapeutic product that is derived from *B. constrictor* is its fat [33]. Treatments for the following ailments are among the applications for this oil: rheumatism, lung disease, thrombosis, boils, tuberculosis, stomach ache, edema, snake bite, cancer, ache, swelling, to prevent abortion, pain, infection, athlete’s foot, calluses, tumours, cracks in the sole of the feet, goiter, earache, sore throat, arthrosis, insect sting, dog bite, erysipelas, asthma, neck strain, and muscle strain [33,107]. Additionally, the species provides a large range of products that are used for many magical/religious purposes, mainly in Afro-Brazilian rituals. Products of the species may also be used to make amulets that are used for various purposes, which include success in love, travel, attracting money, and success at gambling or business dealings [3,4,17]. *Boa constrictor* is among the most preferred snakes to be used as pets because they are beautiful, they are not venomous, and they offer little danger when handled [3]. Its leather is used for ornaments or to produce belts. It also may be killed because it is considered dangerous to the local population, prey birds, or small domestic mammals [3,23].

**Figure 21:**
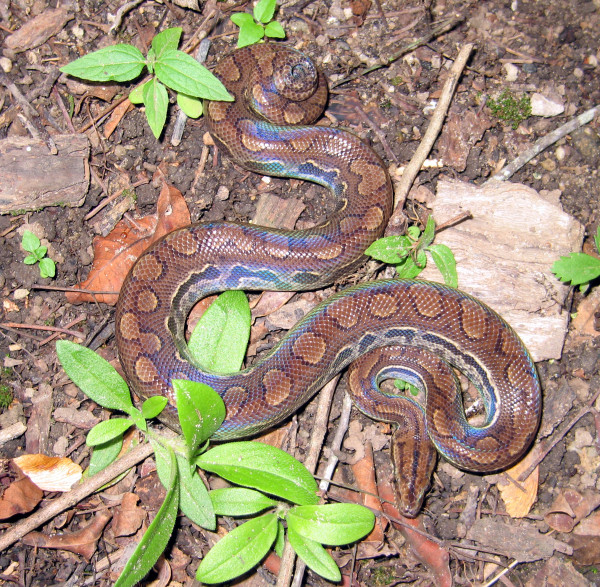
***Epicrates assisi* (Machado, 1945) (Photo – Cláudio Sampaio).**

*Epicrates assisi* (Machado, 1945) (Figure 21) - Common names: Brazilian rainbow boa, “salamanta”.

Comments: This is a medium sized species with a restrict distribution to Caatinga domain [108]. This species is similar to *Boa constrictor* in many ecological aspects. The diet is also based on small mammals; it is a nocturnal species with primarily terrestrial habitats. *Epicrates assisi * presents labial pits and the dorsal ground color presents iridescent pigments. The iridescence distinguishes this species for all others in Caatinga domain.

Ethnozoological notes: This species is often killed due to being confused with poisonous serpents. Its meat can be consumed as food, and its fat is important in popular medicine to treat rheumatism, pain in articulations, self-inflicted injuries and sore throat [33]. The leather is used as ornament or to produce belts.

**Figure 22:**
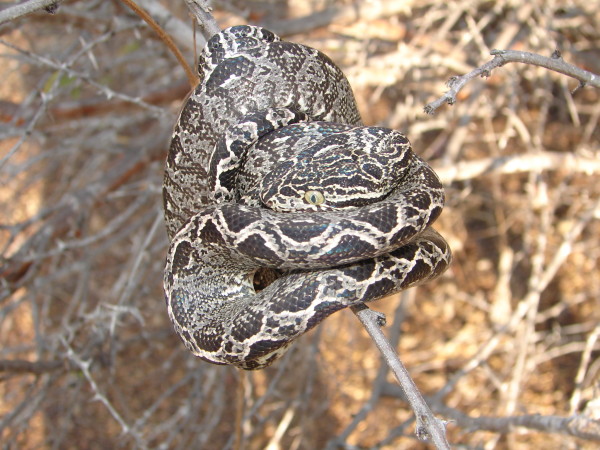
***Corallus hortulanus*(Linnaeus, 1758) (Photo - Washington Vieira).**

*Corallus hortulanus* (Linnaeus, 1758) (Figure 22) - Common name: Tree Boa, “cobra de veado”, “Suaçubóia”.

Comments: Medium sized snake with a wide distribution in Brazil. There are records in Pantanal, Amazônia, Atlantic Forest and Caatinga [109-112]. Rodrigues [112] presents the first record of *C. hortulanus* in Caatinga region specifically to Sao Francisco River sand dunes. There are not others records for this species to Caatinga *censu strictu.* This species is arboreal and nocturnal feeding on small mammals and birds killed by constriction [109,110].

Ethnozoological notes: The species is often killed due to being confused with poisonous serpents. It has been used in popular medicine to treat rheumatism and to help in the removal of spines or other sharp structures from the skin.

#### Viperidae family

**Figure 23:**
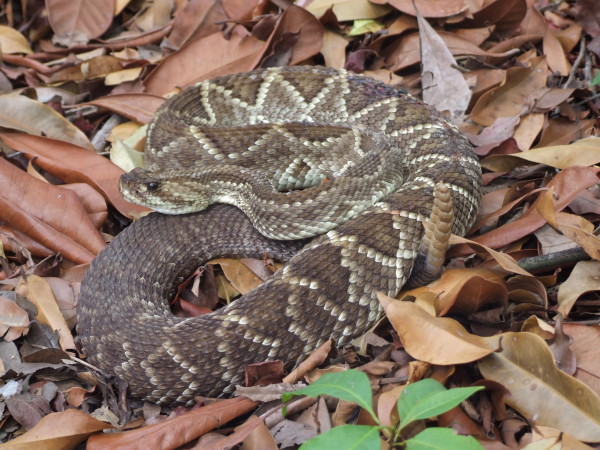
***Crotalus durissus*Linnaeus, 1758 (Photo – Gentil Pereira Filho).**

*Crotalus durissus* Linnaeus, 1758 (Figure 23)- Common names: South American rattlesnake, “cascavel”.

Comments: A medium to large sized species widely distributed from Colombia to Argentina [113]. This species is terrestrial and can be active during day and night. The diet is based on small rodents killed by envenomation. Despite it is considered a species of xeric environments such as Caatinga it is also found in forest environments like Atlantic Forest and Amazon [113]. This species can be easily distinct from all others snakes of semiarid region duo to the tail with characteristic rattle. This rattle is used as a defensive mechanism to avoid potential predators.

Ethnozoological notes: This species is one of the most feared by the inhabitants of the semi-arid region of Brazil. Because of its potential for lethality, it represents a risk for people and domestic animals, which is why members of this species are normally killed when they are found. However, products of this species are used for different purposes. Although it is not typical, in some localities, the meat of the species is consumed as food. Marques and Guerreiro [65] have reported that snake meat snacks were being sold for approximately US$1.50 in the public marketplaces of Feira de Santana, Bahia. The fat of a rattlesnake is used in popular medicine for treating asthma, snake bites, thrombosis, wounds, luxation, rheumatism, pain in the legs, erysipelas, deafness, epilepsy, skin diseases, tuberculosis, hanseniasis, backache, tumours, boils, headaches, earaches, osteoporosis, sore throat, toothaches, for pain relief from injuries caused by insect stings, and for irritation when milk teeth are erupting [33]. The rattle and skin of these organisms are used for magical/religious purposes [17].

**Figure 24:**
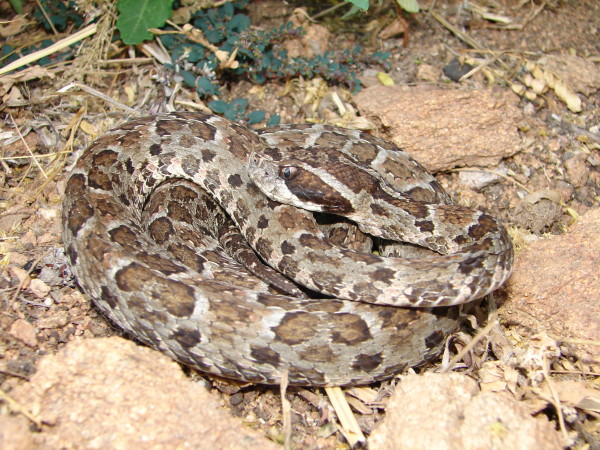
***Bothropoides erythromelas*(Amaral, 1923) (Photo - Washington Vieira).**

*Bothropoides erythromelas* (Amaral, 1923) (Figure 24) - Common names: Caatinga Lancehead, cobra jararaca.

Comments: A small sized lancehead species with a wide distribution within Caatinga domain, considered endemic to this biome [113]. This species is nocturnal and terrestrial, feeding primarily on small mammals, although centipedes and lizards are also consumed. Inhabits semiarid thorn forest, dry deciduous forest and open rocky areas [46].

Ethnozoological notes: This species is feared because of its potential for lethality; its ability to cause the death of domestic animals and people is what motivates its slaughter members of this species whenever they are found. There are no records of products derived from this species that are used by the Caatinga population.

#### Elapidae family

**Figure 25:**
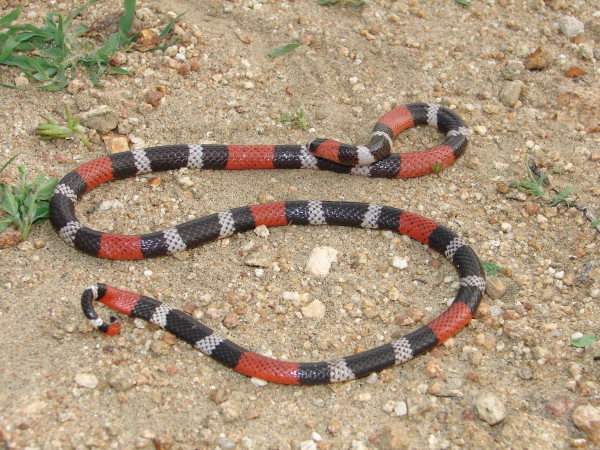
***Micrurus ibiboboca*(Merrem, 1820) (Photo - Washington Vieira).**

*Micrurus ibiboboca* (Merrem, 1820) (Figure 25) - Common names: Coral Snake, “cobra-coral”, Caatinga coral snake.

Comments: A small species with a large distribution in Caatinga domain [113]. This Coral Snake is essentially a terrestrial and nocturnal species, rarely found active during the day. The diet consists on cylindrical preys such as snakes and amphisbaenians killed by envenomation. This species is easily distinct of all others snakes in Caatinga due to the tricolored rings that circulate the body [46]. This species presents a very unique defensive display elevating the tail and performing erratic movements in order to distract and confuse predators.

Ethnozoological notes: This species is usually killed due to representing a risk to people and domestic animals. Medicinal products that are derived from this species are used to treat rheumatism and snake bites [33].

#### Colubridae family

**Figure 26:**
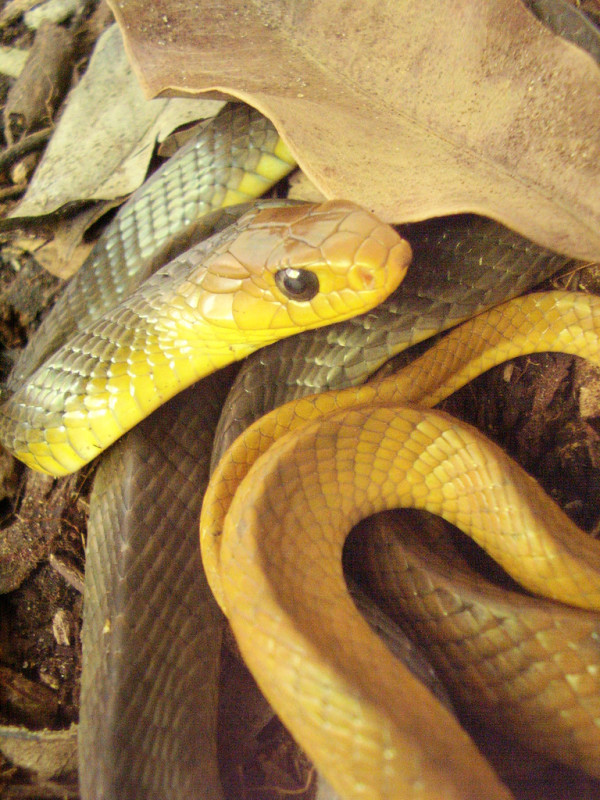
***Drymarchon corais*(Boie, 1827) (Photo - Sanjay Veiga).**

*Drymarchon corais* (Boie, 1827) (Figure 26) - Common name: Indigo Snake.

Comments: This is a medium to large sized snake with a wide distribution in South America, can be found in open (Caatinga and Cerrado) and forest environments (Atlantic Forest and Amazon). This species is diurnal and terrestrial. The diet is generalist including snakes, small mammals, birds, bird eggs and amphibians [109].

Ethnozoological notes: The majority of the time, this species is killed for being thought to be a poisonous animal [13], which is a common reaction to all snakes that are found in the Caatinga.

**Figure 27:**
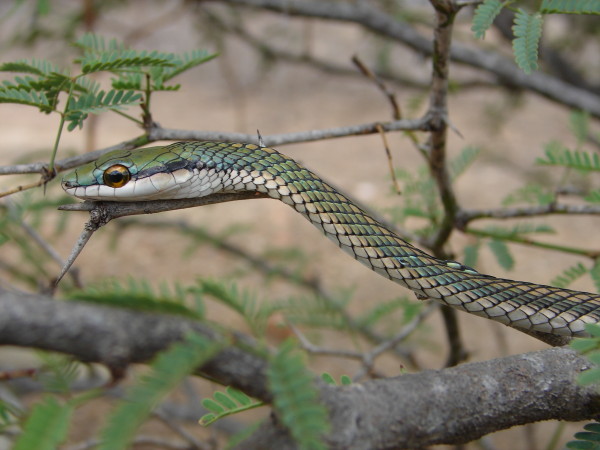
***Leptophis ahaetulla *(Linnaeus, 1758) (Photo - Washington Vieira).**

*Leptophis ahaetulla* (Linnaeus, 1758) (Figure 27) - Common name: Parrot snake.

**Comments:** A small to medium sized snake, distributed in forest and open environments (Caatinga, Atlantic Forest and Amazon) [114]. This species is diurnal and arboreal, the diet is based primarily on amphibians but lizards and small birds are also consumed. *Leptophis ahaetulla* presents a very characteristic defensive display performing false strikes and opening the mouth showing a black mucosa that covers its interior [109].

Ethnozoological notes: Most of the time is killed for being confused as a poisonous animal, a common reaction to all snakes which occur in the Caatinga.

**Figure 28:**
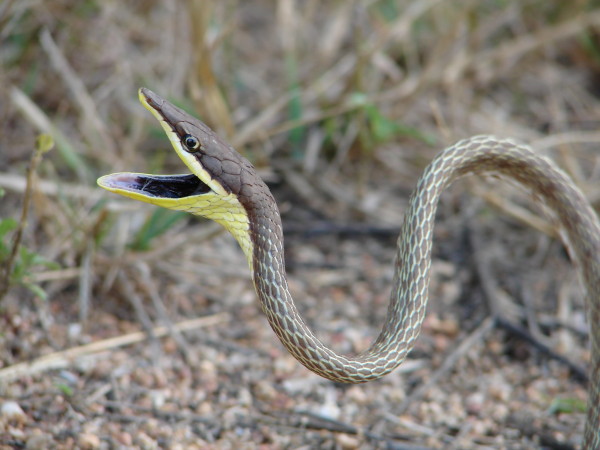
***Oxybelis aeneus*(Wagler, 1824) (Photo - Washington Vieira).**

*Oxybelis aeneus* (Wagler, 1824) (Figure 28) - Common name: Brown vine snake, “cobra cipó”.

Comments: A small to medium sized snake with a large geographic distribution [115]. This species can be found from south Arizona in North America, throughout Central America and most part of South America. In Brazil it inhabits all biomes occurring in open and forest environments. In Caatinga this snakes occurs in all environments. This species is arboreal and diurnal feeding primarily on lizards, but small frogs can also be consumed [109]. The preys are killed by envenomation.

Ethnozoological notes: Most of the time is killed for being confused as a poisonous animal, a common reaction to all snakes which occur in the Caatinga.

**Figure 29:**
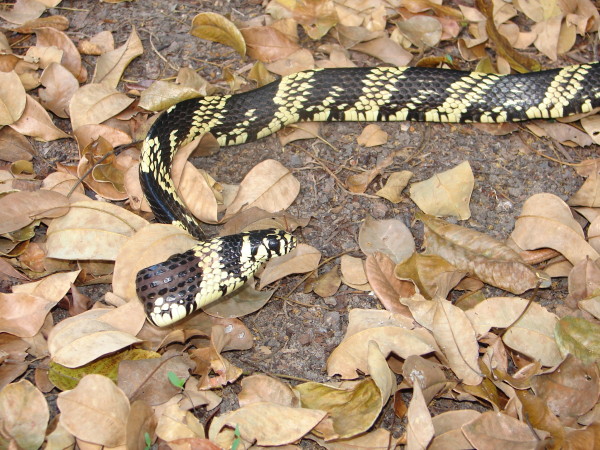
***Spilotes pullatus*(Linnaeus, 1758) (Photo - Washington Vieira).**

*Spilotes pullatus* (Linnaeus, 1758) (Figure 29) - Common names: Tiger snake, caninana.

Comments: A medium to large sized snake with a large distribution in South America, it can be found in Atlantic Forest, Amazon, Pantanal and Caatinga [46,104,109,110]. This species is diurnal with terrestrial and arboreal habits; the diet is generalist consuming a large variety of preys such as mammals, birds, lizards and amphibians [109]. The defensive behaviors consist in biting, performing false strikes, vibrating the tail and inflate the neck region.

Ethnozoological notes: This species is considered to be harmful, and products from this species are used in popular medicine for pain relief from insect stings and snake bites [17].

**Figure 30:**
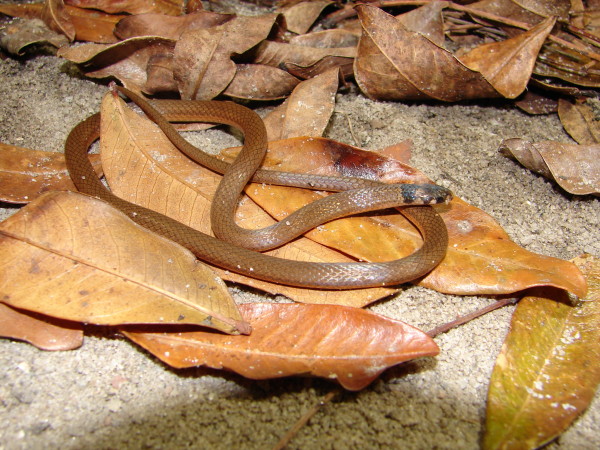
***Tantilla melanocephala*(Linnaeus, 1758) (Photo – Gentil Pereira Filho).**

*Tantilla melanocephala* (Linnaeus, 1758) (Figure 30) - Common name: Black-headed snake, “cobra rainha”, “cobra do folhiço”, Black-headed Snake.

Comments: A small species with widely distributed in Brazil. *Tantilla melanocephalla* can be found in open and forest formations with records in Pantanal, Atlantic Forest, Amazon, Caatinga [46,76,109,110]. This species presents diurnal and nocturnal activity; it is basically a terrestrial and fossorial species. The diet is based only in centipedes killed by envenomation. In Caatinga this species occurs only in forest conditions.

Ethnozoological notes: The majority of the time, this species is killed for being thought to be a poisonous animal.

#### Dipsadidae family

**Figure 31:**
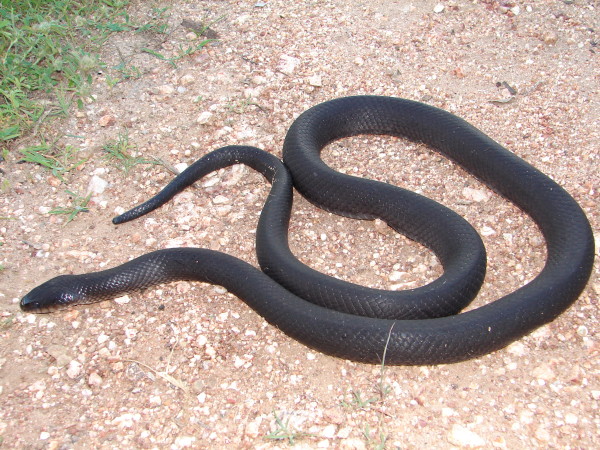
***Boiruna sertaneja*Zaher, 1996 (Photo - Washington Vieira).**

*Boiruna sertaneja* Zaher, 1996 (Figure 31) - Common name: Black snake, “cobra preta”.

Comments: A medium to large species with geographic distribution restricted to Caatinga biome [116]. There is no information about natural history of *Boiruna sertaneja*, although considering the habits of other species of the genus, *B. sertaneja* must have terrestrial habits, nocturnal activity and the diet must be based on snakes and lizards [109]. This species present ontogenetic color change with the juveniles exhibiting coral color in the dorsum and the adults are completely black. *Boiruna sertaneja* is easily distinguished of other black snake of the region *Pseudoboa nigra*. One of the main differences of *P. nigra* and *B. sertaneja* can be observed in subcaudal scales – the subcaudal scales of *P. nigra* are single while the ones of *B. sertaneja* are divided.

Ethnozoological notes: The majority of the time, this species is killed for being thought to be a poisonous animal. However, in some locations, this species is not killed once hunters realise that it is not poisonous and that it eats other snake species, including venomous species [117]. This species is ophiophagous and also eats mammals and lizards [46,118,119].

**Figure 32:**
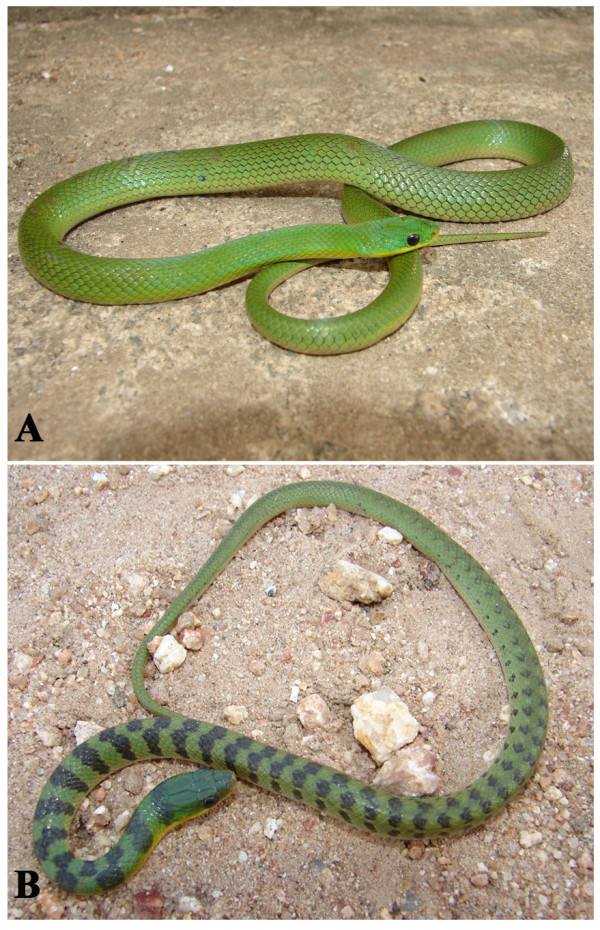
***Liophis viridis*Gunther, 1862.** Adult (above) and juvenile (below) (Photo - Washington Vieira).

*Liophis viridis* Gunther, 1862 (Figure 32) - Common name: Green snake, “cobra verde”, “cobra d’água”.

Comments: A small sized snake with a wide distribution in Caatinga biome [118]. This species is diurnal with terrestrial activity feeding on small frogs and also tadpoles. The juveniles present a green dorsum with lateral black stripes; the adults present a uniform green dorsum. *L. viridis* and *Philodryas olfersii* are the only completely green snakes found in Caatinga, the differentiation of these species is easily detectable in numbers of dorsal scale rows. *L. viridis* present dorsal scale rows in 19-19-17 while in *P. olfersii* the numbers dorsal scale rows are 19-19-15.

Ethnozoological notes: The majority of the time, this species is killed for being thought to be a poisonous animal.

**Figure 33:**
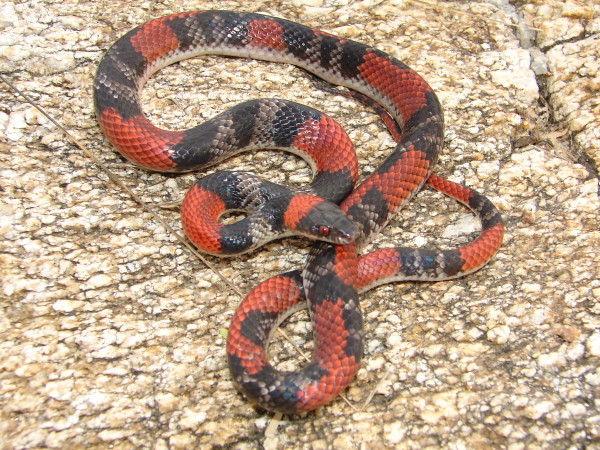
***Oxyrhopus trigeminus*Dumeril, Bibron & Dumeril, 1854 (Photo - Washington Vieira).**

*Oxyrhopus trigeminus* Dumeril, Bibron & Dumeril, 1854 (Figure 33) - Common name: “falsa coral”, “tricolor”.

Comments: A small sized snake distributed throughout Caatinga [46]. The tricolored rings (red, black and white) and the white venter distinguish this species of the others found in Caatinga. Sometimes the venter presents small black spots mainly on cloacal plate. *O. trigeminus* is a nocturnal and terrestrial species; the diet is based on lizards. The preys are killed by envenomation and constriction.

Ethnozoological notes: The majority of the time, this species is killed for being thought to be a poisonous animal.

**Figure 34:**
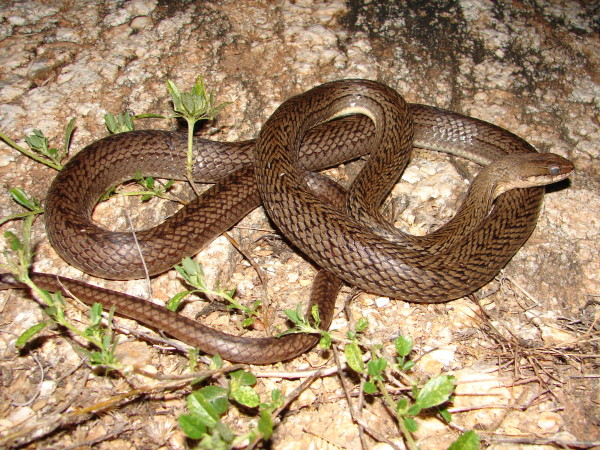
***Philodryas nattereri*Steindachner, 1870 (Figure**33**) (Photo - Washington Vieira).**

*Philodryas nattereri* Steindachner, 1870 (Figure 34) - Common name: “corre-campo”.

Comments: A medium sized snake distributed throughout Caatinga biome [46]. This species is diurnal, terrestrial and feeds primarily on frogs, but sometimes lizards and small mammals can be consumed. This is a very common species in Caatinga; the number of dorsal scale rows of *P. nattereri* in 21-21-17 can distinguish this species of all others in Caatinga.

Ethnozoological notes: The majority of the time, this species is killed for being thought to be a poisonous animal.

**Figure 35:**
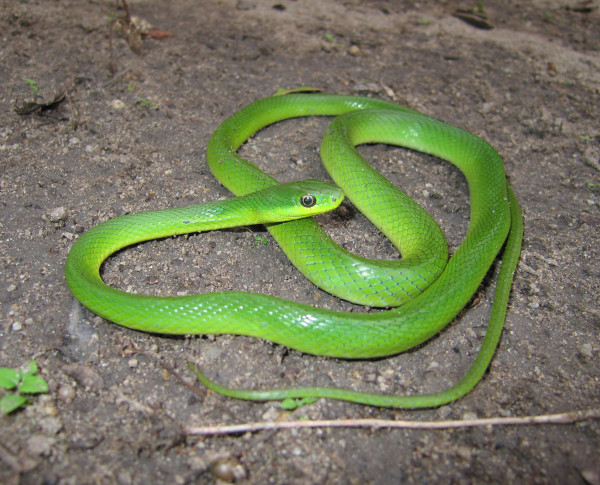
***Philodryas olfersii*(Linchtestein, 1823) (Photo - Washington Vieira).**

*Philodryas olfersii* (Linchtestein, 1823) (Figure 35) - Common name: Green racer, “cobra verde”, Green snake.

Comments: A medium sized snake distributed widely in South America; has been recorded in forest and open vegetal formations such as Atlantic forest and Caatinga [114]. This species is diurnal, terrestrial and the diet is generalist feeding on small mammals, frogs, lizards and birds [118]. The preys are killed by envenomation and constriction.

Ethnozoological notes: The majority of the time, this species is killed for being thought to be a poisonous animal.

**Figure 36:**
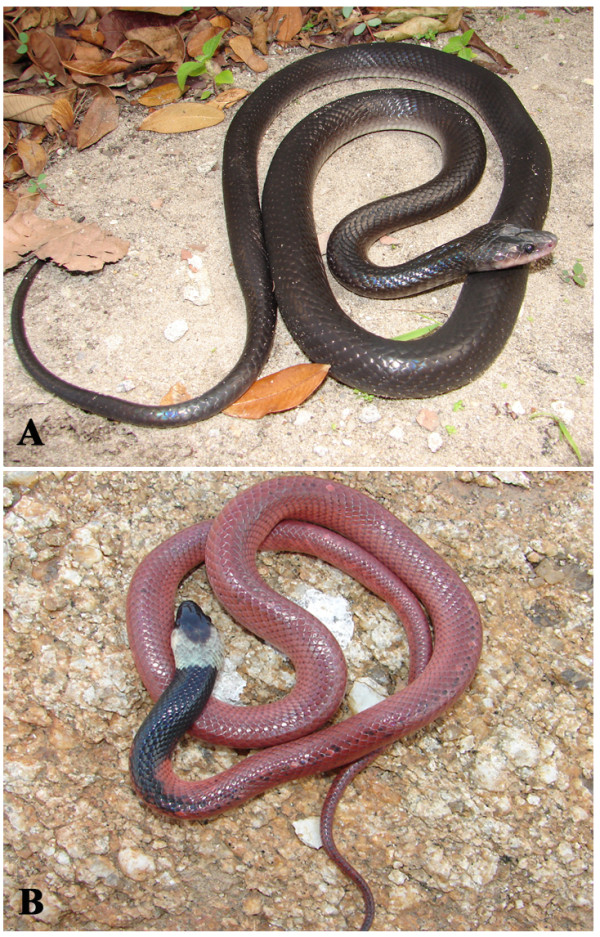
***Pseudoboa nigra*(Dumeril, Bibron & Dumeril, 1854).** Adult (above) and juvenile (below) (Photo - Washington Vieira).

*Pseudoboa nigra* (Dumeril, Bibron&Dumeril, 1854) (Figure 36) - Common name: Black snake, “cobra preta”.

Comments: This is a medium sized snake, distributed in open vegetal formations such Cerrado and Caatinga. In Caatinga biomes it is found in all habitats. This species is nocturnal and terrestrial, the diet is based primarily on lizards but small mammals are also consumed [109]. The preys are killed by envenomation and constriction.

Ethnozoological notes: The majority of the time, this species is killed for being thought to be a poisonous animal.

**Figure 37:**
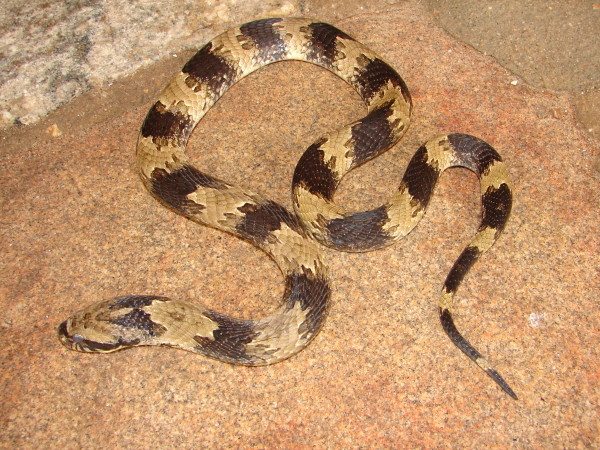
***Xenodon merremii*(Wagler, 1824) (Photo - Washington Vieira).**

*Xenodon merremii* (Wagler, 1824) (Figure 37) - Common name: “focinho-de-cachorro”.

Comments: A medium sized snake with distribution in open vegetal formations such as Caatinga and Pantanal [109,118]. This species is diurnal and terrestrial; the diet is based primarily on toads. This species present a very characteristic defensive display consisting on flattening the region of the neck and open the mouth exhibiting the interior black mucosa. This species often confused with the Caatinga Lance Head *Botrhopoides erythromelas *due to the color of the dorsum, although they are easily distinguish by various aspects [113]. One of the most remarkable aspects is the absence of loreal pits present only in viperids such as *B. erythromelas.*

Ethnozoological notes: The majority of the time, this species is killed for being thought to be a poisonous animal.

### Crocodilians

#### Alligatoridae family

**Figure 38:**
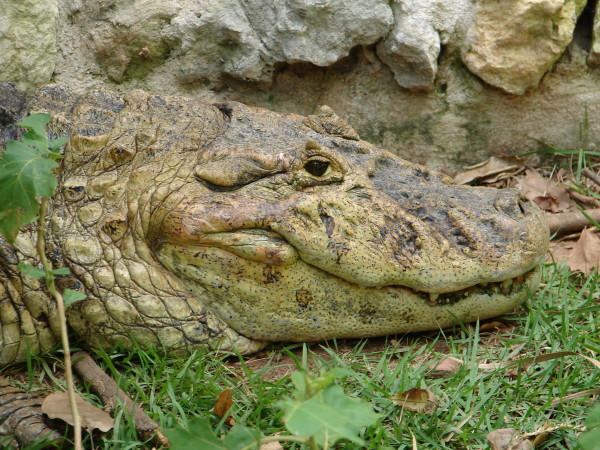
***Caiman latirostris*(Daudin, 1802) (Photo - Washington Vieira).**

*Caiman latirostris* (Daudin, 1802) (Figure 38)- Common names: Broad-snouted caiman,"jacaré-de-papo-amarelo".

Comments: The Broad-snouted caiman is a medium-sized crocodilian that may reach 3.5 m in total length; however, this organism seldom exceeds 2.0 m [120,121]. Its coloration is dark green or greyish with darker spots on the head and neck. Generally, these organisms have three to five dark spots on the jaw, and as it ages, the animal loses its light coloration and becomes completely dark. The womb is yellow-whitish, and young organisms are yellowish with black spots on the lateral portion of the head. The back, tail and iris are greenish [120,122,123]. Its muzzle is nearly as wide as it is long, and it has an infra-orbital crest, two series of post-occipitals, and nuchal shields in three transversal series, with one that is formed by four shields [123,124]. The back has from five to nine shields in the transversal series that are of greater size and ventral shields that are arranged into 21 to 28 transversal series [123,124]. This species is distributed throughout the draining region of the Atlantic Coast of South America (i.e., from the Rio Grande do Norte state, which is the far-eastern area of Brazil, to northern Uruguay) in the draining region of the São Francisco River in northeastern Brazil, in the Paraná River, and in southeastern Paraguay, southern Bolivia and northeastern Argentina [120].

Ethnozoological notes: Humans in the semi-arid region have used *C. latirostris* for food. Its meat is consumed in urban and agricultural areas and is used in the place of the meat of domestic animals. Other products from this species, such as leather and teeth, present other utilities. The leather and fat of the species are used for therapeutic purposes to treat the following illnesses: asthma, sore throat, rheumatism, infection when milk teeth are erupting, hernia and prostate problems, and as amulets for protection against snake bites [66,86,98,101]. The teeth are also used as amulets [86], and the leather can be used to produce handbags and belts.

**Figure 39:**
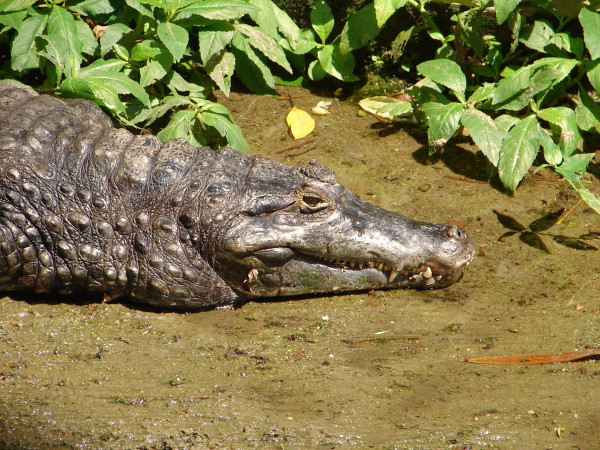
***Caiman crocodilus*(Linnaeus, 1758) (Photo – Rômulo Alves).**

*Caiman crocodilus* (Linnaeus, 1758) (Figure 39) - Common names: Common Caiman, Spectacled Caiman, "jacaré-tinga".

Comments: The spectacled caiman is a medium-sized crocodilian; and the adult specimens may reach a total length of 1.5 to 2.1 m, but they rarely exceed 2.4 m in length. It has a lighter colour than other species of caimans and varies from olive drab with black cross-stripes in adults to yellowish to olive with spots on the flanks and the tail in younger organisms. Occasionally, there is no spot on its jaw [71,122]. It has an infra-orbital crest, and two to four series of small, post-occipital and nuchal shields that are dispersed into four transversal series, where 2 or 3 are formed by four shields [122,124]. Its back has from 8 to 10 shields in the larger-sized, transversal series and ventral shields in the 20 to 24 transversal series [122,124]. It is found to be geographically wide spread in the neotropical region [125,126]. Its occurrence in the semi-arid region of northeastern Brazil has been reported by Borges-Nojosa and Cascon [127] to be in the Serra das Almas Reserve, Ibiapaba city, and in the locality known as Cabaças, which is situated between Ibiapaba and Crateús, Ceará state. The occurrence of this species in these two areas indicates that crocodilians naturally exist in this region and suggests that during the dry period, a population with a wide occupation migrates to dams and temporary lakes.

Ethnozoological note: The uses of this species are similar to those described for *Caiman latirostris*. Its meat is considered to be important, and the fat and leather are used to produce remedies in popular medicine. These remedies have been applied in the treatment of the following ailments: asthma, stroke, bronchitis, backache, earache, rheumatism, thrombosis, sexual impotence, snake bites (as an antidote), evil eye, infection when the milk teeth are erupting, swelling, scratches, ophthalmological problems, sore throat, hernia, prostate problems, and infection and as an amulet to protect against snake bites [66,86,98,101]. The teeth are also used as amulets [86].

**Figure 40:**
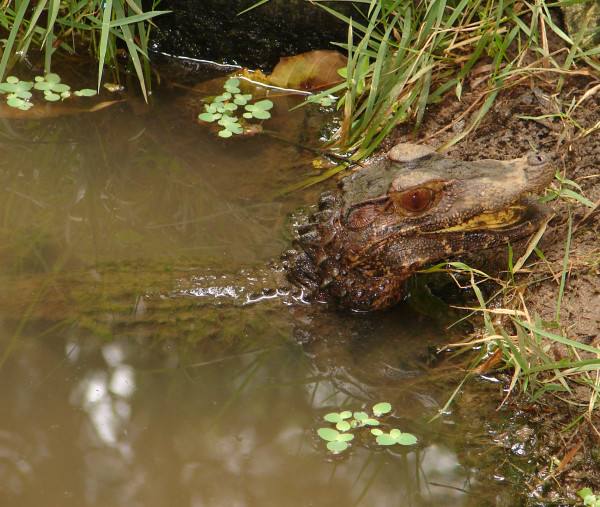
***Paleosuchus palpebrosus*(Cuvier, 1807) (Photo - Washington Vieira).**

*Paleosuchus palpebrosus* (Cuvier, 1807) (Figure 40)- Common names: Cuvier's Smooth-fronted Caiman, Dwarf Caiman, “jacaré coroa”, “jacaré-preto”, “crocodilo”.

Comments: The dwarf Caiman is the smallest species of crocodilian that is found in the neotropical region. Males may reach 1.5 m and females may reach 1.2 m in length [71,123]. The adults are usually dark-brown with large, blackish stripes that spread onto the top portion of the tail. The younglings are yellowish and have dark stripes on their backs. The tail and belly are whitish, and its iris is dark-brown [71,123]. The infra-orbital crest is absent, and there are two series of post-occipital shields; the first one of the two groups has three large shields. The nuchals generally touch each other and are dispersed into four or five transversal series, with two being formed by three or four shields [122,124]. The striking feature in this species is the presence of a crest crown on the posterior region of the head, which is high, short and concave, and a sharp overlapping of the jaw. The armoured nape scales are not as well developed as those of the Smooth-fronted Caiman [71,123]. *P. palpebrosus* is widely spread throughout the neotropical region and is found in the Amazon and Orinoco Rivers, Paraguay-Paraná Rivers basins (except the Pantanal), Atlantic coast drainage areas, São Francisco River, and upper Paraguay River drainage region in Paraguay [128,129].

Ethnozoological notes: The meat of this organism is used as food, and the leather can be useful for producing handbags and belts. In popular medicine, the use of products of this species is directed to treat the following diseases or conditions: asthma, stroke, rheumatism, thrombosis, backache, sexual impotence, edema, mycosis, evil eye, infection when milk teeth are erupting, snake bite (as an antidote), sore throat and as a amulet to protect against snake bite, hernia and prostate problems.

Importantly, the three species of the above-mentioned alligators are occasionally raised as pets, usually when they are young. However, it is not as common to use this group as pets as it is lizards, chelonians and non-poisonous snakes.

### Identification keys for recorded reptiles

Identification key for lizards and amphisbaenia

1. A.  Cylindrical and elongated body (worm-like); limbs are completely absent; small, square scales that are dispersed into rings; and a rhomboid tail........................................2

B.  Morphological features are different from those that are described above.......................................4

2. A.  Pectoral shields are mostly diamond-shaped; head is always dorsoventrally compressed; snout is broad and slightly upturned in the lateral profile; rostrum- and nasal-fused scales, and 2 pre-cloacal pores.........*Amphisbaena polystega*

B.  Pectoral shield is absent; round muzzle; rostrum- and nasal-fused scales; and the number of pre-cloacal pores vary from 4 to 10....................................3

3. A.  Colour of the back varies from yellowish to light brown; whitish belly; round muzzle; a tail without an autotomy level; and 4 to 10 pre-cloacal pores............. *Amphisbaena alba*

B.  Colour of the back is greyish to light brown; whitish belly; short tail with an autotomy level from the fifth to seventh segment; and four pre-cloacal pores ......... *Amphisbaena vermicularis*

4. A.  Absent eyelids; head is covered by granular and small scales; and a back with granular and small scales that are interspersed with tubercles....................................5

B.  Mobile eyelids; top of the head is covered by large scales; the back has scales in various sizes; it may or may not be keeled; and tubercles are absent.......................................7

5. A.  The back has small granules that are interspersed with small, conical and fairing tubercles, and the digits have a double row of lamellae, at least in areas of its extension............ *Hemidactylus mabouia*

B.  Digits with entirely smooth rows of lamellae, and the distal digits are imbricate..............6

6. A.  Greyish or greyish-brown back with 6 to 7 dark brown transverse spots or a reddish-brown and irregular contour that is separated by light grey areas and a yellowish womb in adults................ *Phyllopezus periosus*

B.  The back varies from light grey with dark, transverse stripes that are interrupted by a medium-dorsal light line; a lateral, dark stripe that spans from the orbit to the insertion level of the anterior limb; and a white womb in adults.....................*Phyllopezus pollicaris*

7. A.  Presence of a prominent, vertebral crest, appendix or gular crest, and a large, round scale that is located on the tympanum .................... *Iguana iguana*

B.  Vertebral crest and round scale that is located on the tympanum are absent...................................8

8. A.  Conical-shaped eyes with partially fused eyelids, and the body is relatively slim and laterally compressed ............. *Polychrus acutirostris*B.  Regular eyes and eyelids.....................9

9. A.  Granular dorsal scales that are ventrally large and rectangular...........................10

B.  Dorsal scales may be strongly keeled or small and without a keel, and ventrally smooth and with an irregular shape and size........................................12

10. A.  Adults are usually black and grey or black and dark-brown; either does or does not form transverse stripes; and ventral scales are dispersed in 28 to 34 transverse rows ...................*Tupinambis merianae*

B.  Adults have a variable colour pattern, and ventral scales are dispersed in 8 to 10 transverse rows...........................11

11. A.  Adults are completely green or the anterior part of the body is light brown, and ventral scales are dispersed in 10 transverse rows .................*Ameiva ameiva*

B.  The back of the adults varies from greenish to reddish-brown or has light lines that are interrupted by dark stripes, and ventral scales are dispersed in 8 transverse rows ..................*Cnemidophorus ocellifer*

12. A.  Dorsal scales are strongly keeled, and ventral scales are smaller than those of the dorsal region and are plain, rounded or rhomboid...............*Tropidurus hispidus*

B.  Dorsoventrally flat body; dorsal scales that are small and without keels; and ventral scales are larger than those of the dorsal region...............*Tropidurus semitaeniatus*

Identification key for chelonians

1. A.  High carapace that is dark-brown or black and the areola of the scute is yellow or orange; paws have a columnar shape; and red or orange scales are found on the forelimbs.............*Chelonoidis carbonaria*

B.  Carapace without yellow or orange areola in the shields; paws are not columnar-shaped; and an inter-digital membrane is present...................................2

2. A.  The elongated, oval carapace is high-domed; it has three well-developed, longitudinal keels; the anterior and posterior portions of the plastron are mobile; and it has a retractable neck that moves into the carapace................*Kinosternon scorpioides*

B.  Carapace is slightly flattened dorsoventrally; the anterior and posterior portions of the plastron are mobile; and the neck is retractable and moves laterally near the body Pleurodira.................................3

3. A.  Dark brown or black carapace; the surface of each scute may be roughened with raised striations, a yellowish or completely dark plastron; and the neck is covered with conical tubercles.......*Mesoclemmys tuberculata*

B.  The carapace is dark brown; the plastron is yellowish or with an extensive red and black pattern; the median keel is marked on each side by a series of outgrowths on the costal scute; and the neck has small or fine, long, needle-shaped tubercles....................*Phrynops tuberosus*

Identification Key for snakes

1.  Presence of loreal pits........................ 2

 Without loreal pits............................3

2. A.  Rattle in the end of the tail........................*Crotalus durissus*

B.  Without rattle in the tail.................*Bothropoides erythromelas*

3. Dorsum of the head covered irregularly by small scales...............................4

Dorsum of the head covered by big scales of different sizes.................................5

4. A.  Dorsum of the head covered irregularly by small scales, brown dorsum, without labial pits............................*Boa constrictor*

B.  Dorsum of the head covered irregularly by small scales, presence of labial pits..........................*Epicrates assisi*

5. A.  Small eyes, tricolored rings (red, black and white) ranging from the dorsum until the venter, proteroglyphous dentition...................*Micrurus ibiboboca*

B.  Big eyes without rings..................6

6. A.  With 12 scale rows around the midbody, dorsum with black and yellow colors......................*Spilotes pullatus*

B.  With 15 scale rows around the body.............................7

7. A. With 11 scale rows close to the cloaca......................*Leptophis ahaetulla*

B. 15 scale rows around the body without reduction, brown dorsum with a black helmet.......*Tantilla melanocephala*

C. With 17 scale rows around the midbody.................................8

8. A. 13 scale rows close to the cloaca, extremely elongated head….*Oxybelis aeneus*

B. With 19 scale rows around the midbody.................................9

9. A.  Dorsum with black anterior part and posterior part yellow.................*Drymarchon corais*

B.  Dorsum completely black, divided subcaudal scales.......................*Boiruna sertaneja*

C.  Dorsum completely black, white venter, single subcaudal scales............*Pseudoboa nigra*

D.  Dorsum completely green, 15 scale rows close to the cloaca...............*Philodryas olfersii*

E.  Dorsum completely green, 17 scale rows close to the cloaca....................*Liophis viridis*

F.  Incomplete tricolored rings (red, white and black) in the dorsum, white venter, sometimes with small black and red spots in cloacal and subcaudal region..................*Oxyrhopus trigeminus*

G.  Dorsal keeled scales, dorsum with brown spots, short tail, 2 or 3 post ocular scales..................*Xenodon merremii*

H.  With 21 scale rows around the midbody..............................10

10. A. Dorsum completely brown, white supralabial scales..................*Philodryas nattereri*

Identification key for crocodilians

1. A.  Infra-orbital crest is present ..................2

B.  Infra-orbital crest is absent; 3 large post-occipital shields are found in the first series; a high skull; and a small-sized species.................*Paleosuchus palpebrosus*

2. A.  Greenish iris; muzzle is nearly as wide as it is long; 2 series of post-occipital shields; and the nuchals are dispersed into three series, one of which is formed by 4 shields.....................*Caiman latirostris*

B.  Narrow muzzle that is longer than it is wide; post-occipital shields that are dispersed into 2 to 4 series; and nuchals are dispersed into 4 transversal series and 2 or 3 of these are formed by 4 shields......................*Caiman crocodilus*

## Discussion

Due to the adverse conditions of the environment, a large portion of the human population who live in semi-arid regions have developed a strong relationship with the resources of the local fauna [11,12,27]. Ethnozoologically, reptiles are among the most important vertebrates in Caatinga because of their use or killing due to their conflicting relationships with local people. The cultural importance of these reptiles is also reflected in different myths, legends and beliefs, which, most of the time, affect these animals negatively and therefore, stimulate the fear and aversion to them.

A few reptilian species (n = 13) are used as food in the semi-arid region, for example, the large-sized lizards and snakes. The lizard *T. merianeae* is the main species with food value. Although it is not common, other reptiles can also be eaten, such as *B. constrictor, I. iguana* and *C. durissus*. Nonetheless, the main, practical value of reptiles appears to be in popular medicine, where the products of several species of chelonians, snakes and lizards are recorded to be used as remedies for illnesses by the local population. This situation is not surprising because reptiles are among the most frequently used species in Brazilian traditional folk medicine [33,39,86,130-132]. *Tupinambis merianae*, *C. durissus*, *I.iguana *and *B. constrictor* are the most common reptile species to be used for medicinal purposes in the semi-arid region. Particularly, *T. merianae* and *B. constrictor* are used because of their wide applicability in traditional Brazilian medicine [4,33,133]. In the northeastern region, products from these species are used in traditional communities and are commercialised in public marketplaces in many cities [17,34,38,41,66,98,106]. Recent works have investigated the use of the fat of these two species, and these studies have shown that these products may be effective for some illnesses [134-136].

Different sub-products from the recorded reptiles may also be used for magical, religious and ornamental purposes and to produce handbags and belts. Mainly, non-perishable products, such as the leather of lizards and snakes and the carapace of chelonians, are used for these purposes. As was observed by Moura and Marques [99], the use of these types of sub-products can be justified as an attempt to maximise the resources from the local ecosystems. In fact, ethnozoological studies have shown that animal parts that are improper for alimentary consumption (such as leather, teeth, carapace and skulls) are used for medicinal and magical/religious purposes and to produce ornaments or souvenirs [137-145].

Despite the utilitarian value of many species of reptiles that are found in the Caatinga, a large number of these animals are killed for being considered harmful to people and domestic animals. From the 38 species that were recorded in this study, 19 (50%) are involved in a conflicting relationship with the local population, especially the serpents. Furthermore, the number of species that are associated with these types of conflicts is larger because 52 species of serpents, the group that is generally most feared and hunted by the local population, are registered in the semi-arid region. The main victims of accidents with snakes are the agriculturists who contact the serpents during their practices, and as discovered by Vizotto [5], the Brazilian agricultural population tends to consider all serpents as poisonous, which a perception that has spread in the northeastern semi-arid region. Therefore, it is presumed that all serpents are subject to harassment by the local population. Additionally, the amphisbaenia is included in the undesirable list of animals because it has a similar morphology to serpents [3,68,146,147].

The aversive feelings for serpents are justified by the lethality risk that some species offer and motivate the indiscriminate slaughter of these animals in the semi-arid region [11]. Between the years 2000 and 2009 in northeastern Brazil, 71,055 cases of snake accidents and 394 deaths from snake bites were reported [148]. Only a small portion of the snake accident cases that were reported resulted in the death of the victims. However, the oral stories of accidents and deaths that were related to serpents contributed to the spread of fear of these reptiles. Because snake accidents can involve any serpent, the possibility of occurrences that are caused by non-poisonous species is high. Importantly, of the 52 serpent species that are found in the northeastern, semi-arid region, only four species are venomous. However, every snake that is found is usually killed, independent of its potential for lethality. According to Fernandes-Ferreira et al.[7], the human perception about dangerous situations involving animals may be not directly linked to the actual threat level that these animals present. In this context, in the northeastern semi-arid region, the conflict between people and serpents are related culturally and by the lack of knowledge about the serpents regarding the occurrence of deaths that are caused by these reptiles.

Because the human population who lives in the Caatinga usually does not know if a serpent is venomous, all serpents are usually killed when found. The same aversion to reptiles that was observed in this work has been recorded in places in Brazil. Moura et al. [22] have found that, in several regions of Brazil, there is an acceptance of a negative stereotype for all serpents, which are generally considered to be "dangerous animals". This situation, which is associated with some aspects of popular culture, can potentiate the conflicts between human beings and serpents [3,149,150] and negatively influence how people interact with animals from this group [5,151].

Although reptiles are killed because of this conflicting relationship, sub-products of these slaughtered animals may be used in different ways by local inhabitants. Thus, a paradoxical situation occurs in that the products of a dead species, although it is considered harmful, can be used by people. For example, the sub-products of “teiús” (*T. merianae*), which are commonly killed to prevent them from eating eggs and chicks, are used as ornaments and in popular medicine [34,36,152,153]. Venomous snakes, such as the rattlesnake (*C. durrisus*), coral (*M. ibiboboca*) and jararaca (*B. erythromelas*), are killed as a precaution; therefore, while they can attack domestic animals or people, and their sub-products (e.g., rattle or fat) are used for mainly medicinal purposes [86].

As discussed before, the interactions of the population with reptiles that are found in Caatinga are varied, and such relationships have obvious implications for the conservation of the herpetofauna from this biome. In this context, ethnoherpetology studies are crucial because they serve as subsidies for guiding strategies for the handling and conservation of reptiles in different areas.

The interaction between people and reptiles has been studied in Caatinga [32], but is restricted to only a few localities and States. Another problem is that many ethnozoological studies that are carried out in Brazil and other countries highlight only the vernacular names or the identification of a species is made through “*taxonomic clues*” [e.g. [154-156], which is an alternative resource for obtaining information. Often, this method can induce serious taxonomic mistakes [157]; therefore, it must only be used when a species cannot be adequately identified and as a last resource.

The need for greater taxonomic severity in ethnozoological studies has been emphasised [32], and suggests that researchers collect samples of specimens for every species that is recorded in their research. This method will allow for standards of use, and the interactions between people and other animal species in different localities of the semi-arid region can be confirmed by ethnozoologists, taxonomists, and biologists.

The importance of voucher specimens in ethnobiological investigations has been repeatedly emphasised [158,159]. A well-developed ethnozoological study, which includes a collection of voucher material, contributes to the quality of the research and can subsidise zoological research in all related themes (e.g., taxonomy, zoological inventories and biogeography). This method can also enable the recording of new species and the broadening of the geographic distribution of previously described species [157]. Silitoe [160] related the discovery of the hilideo, *Litoria bulmeri*, to an ethnoherpetological study by the anthropologist Ralph Bulmer, who was honoured by the nomination of the species. This author highlights that, in his research, which was carried out in New Guinea, he collected a new species of micro-hilideo (*Choerophryne sp*.) that was not formally described because only one specimen was found. Several authors have highlighted the role of ethnozoology as an important tool for making inventories and enriching ecological surveys [11,31,161].

Because different interactions between people and reptiles in the northeastern semi-arid region may have ecological implications on the natural populations of those species, there is an urgent need to implement actions that can control the interactions that negatively affect the involved species. Furthermore, these actions must take into account the cultural, economic, social and ecological aspects of the local human populations. The use of wild animals must be directly addressed, and educational programs that are directed towards all associated actors should be initiated. Environmental education programs that aim to create an environmental, national culture that supports the protection of these species could potentially change the attitudes of people regarding those reptiles. As pointed by Alves et al. [162], these programs could be carried out locally by trained community organisers in areas where the species are more exploited or through environmental education programs that are linked to Brazil’s national curriculum. This idea is particularly feasible because public schools are organised nationally by the federal government [162]. The implementation of activities of environmental education can increase the awareness of the importance of snakes, instructing those who still consider them intrinsically harmful [3,22]. Poughet al. [163] emphasize that human education is urgently needed at all levels to help maintain viable populations of reptiles. Training in areas such as habitat protection, wildlife management, and conservation biology is needed — especially in tropical countries where most reptile species are found. The success of conservation and management programs ultimately depends on how well these programs are tailored to the interests and needs of the people where the threatened and/or endangered animals live.

Several factors have contributed to the decline of reptile populations in Brazil, and the direct consumption of these animals and negative interactions because of conflicts are only a part of the larger problem [3,4]. In the semi-arid region, non-sustainable human activities, such as slash and burn agricultureand the continuous use of native pastures for goat and cattle, are causing huge-scale environmental impoverishment in the Caatinga biome [164]. In dry regions, climate change will also interact with human activities to increase the risk of desertification [49,165]. The hunting of wildlife must be considered an anthropogenic pressure, like the loss of habitat [11,12]. The extinction of species and the reduction in the number of species has ecological consequences [166]. Because the reduction or local extinguishing of a species can result in a series of serious, negative impacts that mainly occur in the food chain [167-169]. A decrease in the populations of serpents can generate uncontrolled populations of rodents, which could cause plagues and damage the health of human beings, agriculture and the wild-food chain [7]. Furthermore, the slaughter of ophiophagous serpents may cause an increase of the population of poisonous snakes [170].

Strategies for conserving the herpetofauna of the Brazilian semi-arid region must reconcile and integrate human and conservation needs. Within this context and to achieve sustainability in the use of wild species, it is necessary to establish which species are being captured and to understand the socioeconomic and cultural significance of their use and/or commercialisation. Because a wide variety of threats to reptiles exist, education and enforcement programmes must be combined with programmes that can protect wildlife habitats, which will benefit reptiles and other animal groups.
